# The Knowledge Base for Achieving the Sustainable Development Goal Targets on Water Supply, Sanitation and Hygiene

**DOI:** 10.3390/ijerph13060536

**Published:** 2016-05-27

**Authors:** Guy Hutton, Claire Chase

**Affiliations:** 1 United Nations Children’s Emergency Fund (UNICEF), New York, NY 10017, USA; 2 Water and Sanitation Program, The World Bank, Washington, DC 20433, USA; cchase@worldbank.org

**Keywords:** water, sanitation, hygiene, health, nutrition, cost-effectiveness, cost-benefit, economic analysis, environment, water security

## Abstract

Safe drinking water, sanitation, and hygiene (WASH) are fundamental to an improved standard of living. Globally, 91% of households used improved drinking water sources in 2015, while for improved sanitation it is 68%. Wealth disparities are stark, with rural populations, slum dwellers and marginalized groups lagging significantly behind. Service coverage is significantly lower when considering the new water and sanitation targets under the sustainable development goals (SDGs) which aspire to a higher standard of ‘safely managed’ water and sanitation. Lack of access to WASH can have an economic impact as much as 7% of Gross Domestic Product, not including the social and environmental consequences. Research points to significant health and socio-economic consequences of poor nutritional status, child growth and school performance caused by inadequate WASH. Groundwater over-extraction and pollution of surface water bodies have serious impacts on water resource availability and biodiversity, while climate change exacerbates the health risks of water insecurity. A significant literature documents the beneficial impacts of WASH interventions, and a growing number of impact evaluation studies assess how interventions are optimally financed, implemented and sustained. Many innovations in behavior change and service delivery offer potential for scaling up services to meet the SDGs.

## 1. Introduction

Safe drinking water, sanitation, and hygiene (WASH) are fundamental to an improved standard of living, including the protection of health and the environment, improved educational outcomes, greater convenience, dignity and gender equality. Poor and vulnerable populations have lower access to improved water, sanitation, and hygiene (WASH) services and poorer associated behaviors. Improved WASH is central to reducing poverty, promoting equality, and supporting socioeconomic development. For these reasons, drinking water and sanitation were included as targets in the Millennium Development Goals (MDGs) and aspirations for the post-2015 period under the Sustainable Development Goals (SDGs) are to achieve universal access to WASH by 2030. Furthermore, the Human Right to Safe Drinking Water and Sanitation (HRTWS) was adopted in 2010 under a United Nations (UN) Resolution calling for safe, affordable, acceptable, available, and accessible drinking water and sanitation services for all [1].

As the world moves into the post-2015 period, in addition to enhancing monitoring efforts, greater understanding is needed of the challenges facing the world to meet universal access within 15 years and sustain it beyond 2030. Unsustainable water extraction together with competing demands, population growth and migration (including urbanization), water pollution from release of untreated sewage and wastewater, climate change and climate variability all add very significant pressures on water supply systems, as well as require systematic, coordinated planning of new settlements and retrofitting of existing settlements to deliver sustainable water services. And as countries approach universal access to improved sanitation, further investments are needed to reduce the release of untreated fecal waste into the environment and exploit it for its energy and mineral content. The economic impacts of lack of access to WASH services are still very high, including the health, environmental and social burdens. To make the most use of available resources it is critical to have the best available knowledge on, for example, the current WASH coverage, the impacts of inadequate WASH and the WASH interventions that work at scale in order to define and implement the most cost-effective WASH policies and interventions.

## 2. Materials and Methods

The aim of this paper is to summarize global evidence on WASH and to recommend future focus areas for research and policy. The paper covers evidence showing progress in drinking water, sanitation, and hygiene coverage; impacts of poor WASH, covering health, social, environmental and economic aspects; evidence on the effectiveness of WASH interventions; and the costs and socioeconomic returns of improved WASH. The scope of WASH services included is shown in Table 1. The focus is on services at the household and institutional level and for personal rather than productive uses.

The review followed a structured search process using: thematic terms covering costs, damages, effectiveness, impacts, benefits, policy, financing, monitoring; impacts covering health, environment, social, economic and financial; intervention terms on water, sanitation and hygiene (see Table 1); and geographical terms covering developing countries. Guided by the topic areas outlined above, evidence was sourced mainly from published synthesized reviews, such as systematic reviews, meta-analyses and literature reviews. When these were not available, evidence was compiled from the next best sources of published research. Evidence was critically assessed to determine the quality of methods and robustness of results using accepted hierarchy of evidence criteria for health effectiveness studies. Unpublished and grey literature was used where no peer-reviewed published evidence exists.

## 3. Results

### 3.1. Status of Drinking-Water, Sanitation, and Hygiene

#### 3.1.1. Targets

The MDG 7c targets called for halving the proportion of the population without sustainable access to safe drinking water and basic sanitation between 1990 and 2015. The targets were ambitious at the outset, with 76% of the population globally using an improved drinking water source and 54% with access to safe sanitation in 1990. The drinking-water target was met in 2010, while in 2015 the world remains 9% points short of achieving the sanitation target. The Sustainable Development Goals (SDG) for the period 2015 to 2030 have broadened to include efficient water resource allocation and use, and integrated water resource management, as well as setting ambitious WASH-related targets of universal access to safe water (Target 6.1), adequate sanitation and hygiene, and eliminating open defecation (Target 6.2) and reducing untreated wastewater (Target 6.3). Within the overall aim of access for all, the language and spirit of the SDGs emphasizes the reduction of inequality and the provision of inclusive, quality and sustainable services—ensuring access for women and poor and vulnerable populations.

#### 3.1.2. Definitions

To understand the status of drinking water, sanitation, and hygiene, a distinction is necessary between different levels of service access and population practices. While all populations meet their water and sanitation needs in some way, it is often not sufficient, reliable, safe, convenient, affordable or dignified. Hence, for MDG monitoring there was a distinction between “improved” and “unimproved” water and sanitation facilities at home. For the SDG targets, one indicator has been proposed per target: “percentage of population using safely managed drinking water services” (Target 6.1); and “Percentage of population using safely managed sanitation services including a hand washing facility with soap and water” (Target 6.2). Feeding into these proposals was a broader set of indicators distinguishing ‘basic’ and ‘safely managed’ service levels, shown in Table 2 [2].

While indicators for global monitoring need to be kept simple for reasons of feasibility and cost, countries, organizations, and projects often monitor different aspects of service performance, such as quantity, quality, proximity, reliability, price, and affordability [5]. Some countries adopt more lenient definitions than those of the JMP, and some adopt stricter definitions.

The definitions used in existing monitoring systems have several limitations. First, the JMP’s definition of improved access focus on the technology type is an imprecise proxy for the quality of the services [6,7,8]. Second, the self-reported responses of access by household members may have an upward bias [9]. Third, statistics on household access provide no indication of variation in access and practices among different household members. For example, even in communities with high sanitation coverage rates, children still commonly defecate in the open. Fourth, the MDG indicators do not reflect well accountability and sustainability. The existing approach to measuring access does not provide a good indication of sustainability as the surveys use representative sampling and do not follow individual households over time. The new indicators for safely managed services, shown in Table 2, were informed by the five normative criteria of the Human Right to Safe Drinking-Water and Sanitation (HRTWS): accessibility, acceptability, availability, affordability, and quality [10]. For water supply, the first level of water supply service reflects the MDG “improved“ water indicator except that the former excludes water sources with greater than 30 min round trip. For sanitation, the first level of sanitation service reflects closely the MDG “improved” sanitation indicator. For both water supply and sanitation, the SDG Targets 6.1 and 6.2 refer to a higher level of service: “safely managed”. However, given that many countries have still not achieved universal coverage of this first level of service, it will continue to be monitored and reported by the JMP. In the SDG period, additional questions will need to be added to the existing questions on WASH access, or else the use of household survey data for measuring household access will therefore need to be complemented with additional surveys to understand quality aspects, intra-household variation and rates of facility break-down or non-use.

The following sections present the coverage data at global and regional levels for drinking water and sanitation according to the JMP definitions used for monitoring MDG Target 7C, using the most recent update and MDG assessment report [11]. 

#### 3.1.3. Coverage of Water Supply

**Figure 1:**
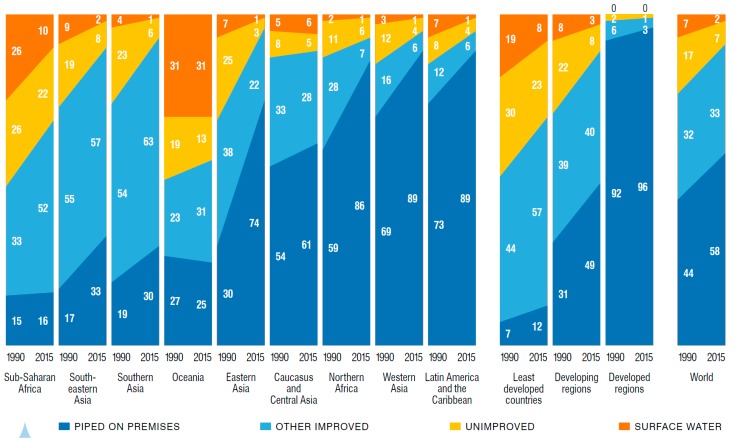
Drinking-water coverage trends by developing regions and the world, using the JMP improved water definition, 1990–2015. Reproduced with permission from World Health Organization and UNICEF [11].

Globally, the use of improved drinking water sources increased from 76% in 1990 to 91% in 2015 [11]. Regional breakdowns for progress between 1990 and 2015 are shown Figure 1. In its 2012 report presenting 2010 estimates, the JMP announced that the world had met the MDG target of reducing by 50% the population without access to safe drinking water [12], but these global estimates mask regional disparities and inequities in access between urban and rural populations. As of 2015, 663 million people still use unimproved water sources (compared to 1.3 billion in 1990), while 2.6 billion people have gained access to improved water since 1990. Sixteen percent of rural dwellers remain unserved, compared with 4% of urban dwellers. In Sub-Saharan Africa, 44% of rural dwellers continue to use an unimproved water supply. Water hauling costs Africans, especially African women, billions of hours of their time every year. In 2008, for example, more than a quarter of the population in several Sub-Saharan African countries takes longer than 30 min to make one round trip to collect water; 72% of the burden for collecting water falls on women (64%) and girls (8%), compared with men (24%) and boys (4%) [13].

Urban areas enjoy a higher level of water service, as indicated by the use of piped water supply; in 2015, four out of five people living in urban areas uses piped water, compared to just two out of three in rural areas. Water sources classified as improved, even piped water, do not guarantee the safety or continuity of the water supply. Water quality surveys conducted in five countries showed microbiological compliance with the World Health Organization (WHO) guidelines that varied between water sources and countries [8]. While thermotolerant coliform (TTC) count was positively associated with sanitary risk on a scale of 0 to 10 for both piped water supply and other improved sources, TTC counts of above 10 cfu/100 mL were still common especially for other improved water sources. Extrapolating to global estimates, the authors estimate that in 2010, 1.8 billion people (28%) used unsafe water, more than twice the 783 million population (11%) that used an unimproved water supply.

#### 3.1.4. Coverage of Sanitation

**Figure 2:**
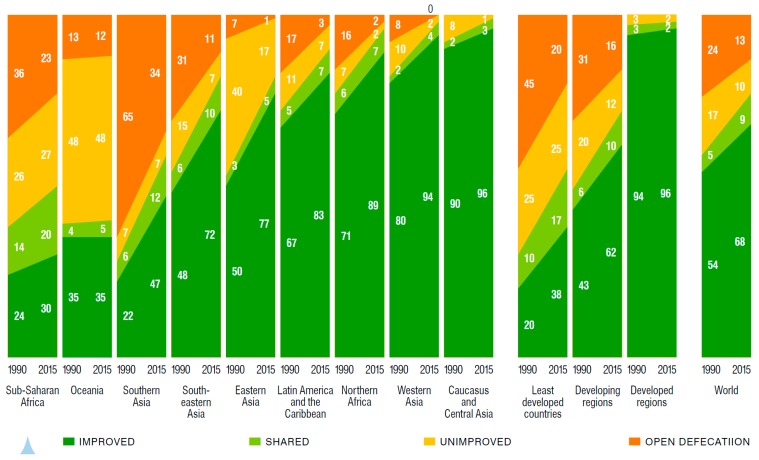
Sanitation coverage trends by developing regions and the world, using the JMP improved sanitation definition, 1990–2015. Reproduced with permission from World Health Organization and UNICEF [11].

The use of improved sanitation has increased from 54% in 1990 to 68% in 2015, 9% points short of meeting the global MDG target [11]. In 2015, 2.4 billion people still did not have access to their own improved sanitation facility, which reflects no change on the population unserved in 1990 due to population growth. These numbers mask that since 1990 2.1 billion people have gained access to improved sanitation. Regional statistics on progress between 1990 and 2015 are shown in Figure 2. Globally, the proportion of population practicing open defecation declined from 24% in 1990 to 13% in 2015. In South Asia, 34% of the population still defecates in the open, compared with 23% in Sub-Saharan Africa. Globally, 638 million people (9%) share their sanitation facility with another family or families. Comparing rural and urban areas, 51% of rural dwellers have access to improved sanitation, compared to 82% of urban dwellers. Rates of ‘improved’ sanitation do not reflect the amount of fecal waste that is not isolated, transported or treated safely—a study of 12 cities in low- and middle-income countries found that while 98% of households used toilets, only 29% of fecal waste was safely managed [14].

#### 3.1.5. Coverage of Hygiene

Although the MDG Target 7C does not provide a global indicator for hygiene, the data on the presence of a handwashing facility with soap and water present are increasingly collected as part of nationally representative surveys, and will form the basis for efforts to monitor Target 6.2 of the SDGs. Research studies suggest that the global prevalence of handwashing with soap after contact with excreta is 19%, with lower rates in Sub-Saharan Africa (14%) and Southeast Asia (17%) where the most studies have been conducted [15]. Proxy indicators for handwashing practice from nationally representative surveys (for example, self-reported handwashing or the presence of a handwashing station) are not reliable indicators of actual behavior, and tend to over report hygiene practices [16].

#### 3.1.6. Distribution of Services

**Figure 3:**
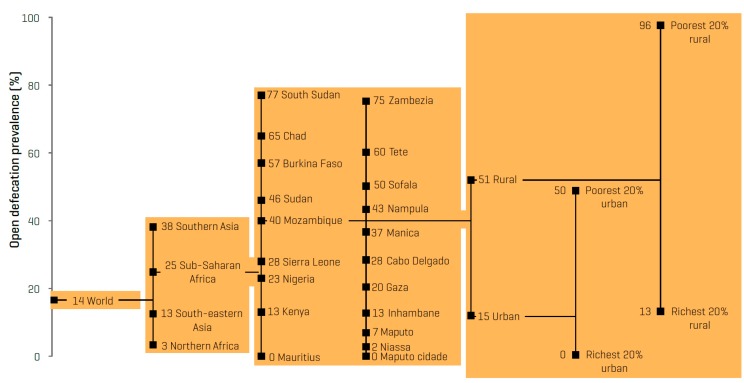
Example from Mozambique on how average access values mask massive disparities in household coverage. Reproduced with permission from World Health Organization and UNICEF [18].

The JMP has reported the distribution of water supply and sanitation services by wealth status, breaking the population into five equal wealth quintiles using an asset index. In 35 Sub-Saharan African countries, for example, households in the poorest wealth quintile are six times less likely to have water access, compared to the richest quintile; the difference for sanitation is at least 2.5 times [17]. Figure 3 illustrates the different levels of disparity across regions, countries, at a sub-national level and between wealth quintiles. Limited data sets are available on the disparities between population subgroups—for example, slum populations, ethnic groups, women, elderly, and persons who are physically impaired—as the sample size and sampling methodology in nationally representative surveys generally do not enable sufficiently robust comparisons.

#### 3.1.7. Institutional WASH

Global reporting of institutional WASH has not yet been standardized, and efforts are now underway to build a global reporting system of WASH in schools and health facilities for SDG monitoring. The Demographic and Health Survey (DHS) Service Provision Assessment (SPA) collects data on WASH in health facilities. WASH coverage in both primary schools and front-line health facilities is monitored and reported under the Service Delivery Indicators, currently for Sub-Saharan Africa. United Nations agencies collect data on WASH in schools (Education Management Information System, operated by UNICEF), health facilities (Health Management Information System, operated by the WHO), and refugee camps (Office of the United Nations High Commissioner for Refugees (UNHCR)).

### 3.2. Impacts of Inadequate WASH

Understanding the nature and extent of the negative impacts of inadequate WASH on individuals, the environment, and societies is important for those designing interventions and assessing benefits and efficiency. Indeed, many benefits of WASH interventions are non-health in nature and hence only including health impacts in impact evaluations can severely underestimate the intervention benefits [19]. Hence later sub-sections focus on impacts on well-being, the environment and financial and economic consequences.

#### 3.2.1. Health Consequences

Contaminated water and lack of sanitation lead to the transmission of pathogens through feces, and to a lesser extent, urine. The F-diagram provides a basic understanding of these pathways, where pathogens from feces are ingested through transmission via fingers, flies, fluids, fields (soil) and food. Diseases transmitted via the fecal pathway are diarrheal disease, enteric infection, hepatitis A and E, poliomyelitis, helminthes, trachoma, and adenoviruses (conjunctivitis) [20]. Most of these diseases are transmitted through the fecal-oral pathway, but some are through fecal-skin (for example, schistosomiasis) and fecal-eye pathways (for example, trachoma). Trachoma is endemic in more than 50 countries—in 2004, about 1.3 million people were estimated to be blind from trachoma and probably a further 1.8 million have low vision, and causing about 1.3 million Disability-Adjusted Life Years (DALYs) [21]. These transmissions occur between humans, as well as between animals and humans. Pathogens carried via urine are mainly those involved in animal-to-human transmission, such as leptospirosis. Poor personal hygiene causes fungal skin infections, such as ringworm (tinea) and scabies. Lack of handwashing is associated with respiratory infections [22], while inadequate hand hygiene during childbirth is linked to infection [23] and neonatal mortality [24,25]. Poor water and sanitation is also linked to maternal mortality [26].

Water availability for drinking and household uses can affect the quantity of water consumed and the time available to care for children in the household. Reducing the distance required to fetch water is associated with lower prevalence of diarrhea, improved nutrition, as well as reductions in under-five child mortality [27], possibly because it enables better hygiene practices [28] and frees up time for child care or income generating activities [29], resulting in healthier children.

Inadequate quantities or consumption of water can also lead to dehydration which has a number of adverse impacts on physical and cognitive performance, as well as bodily functions [30]. However the effect remains undocumented due to lack of adequate biomarkers to measure hydration status at the population level [30]. Furthermore, safe drinking water provides the basis for oral rehydration solutions that save lives [31].

Exposure to harmful levels of arsenic in groundwater is estimated to impact 226 million people in more than 100 countries [32] causing skin lesions and long-term illnesses such as cancer, neurological disorders, cardiovascular diseases, diabetes, and cognitive deficits among children [33].

Excess levels of water through heavy rainfall and inadequate drainage leads to flooding, causing injuries and death, as well as heightened risk of fecal-oral and skin diseases [34]. Earthquakes, volcanic eruptions, tsunamis and other natural disasters leave affected populations vulnerable to infection with waterborne diseases such as diarrhea, hepatitis A and E and leptospirosis [35].

##### Diarrheal Disease

The most recent study estimated a total of 842,000 global deaths from diarrheal disease for 2012 [36]—43 percent of these in children under five years of age. An estimated 502,000 deaths were caused by inadequate drinking water; 280,000 by inadequate sanitation; and 297,000 by inadequate hand hygiene (Table 3). The regional breakdowns indicate that the major share of global burden is in Southeast Asia and Sub-Saharan Africa. The Global Burden of Disease study recently conducted a new meta-regression analysis of experimental and quasi-experimental interventions, estimating 542,000 fewer deaths than the WHO study attributable to poor water and sanitation. The Global Burden of Disease study did not account for different levels of quality of water supply and sanitation, and between poor quality implementation and lack of effect, which may partially account for the difference.

Not all diarrheal diseases are caused by pathogens transmitted via inadequate WASH. Over time, different estimates have been made for the burden of diarrheal disease attributable to fecal-oral transmission. Earlier estimates use an attributable proportion of diarrheal disease due to poor WASH of 88% [37], while the more recent study used 58% [36]. This latter estimate is closely supported by a separate review of over 200 studies that examined the causes of diarrhea in inpatients and found that no pathogen was present in 34% of cases [38]. Importantly, deaths not easily preventable through WASH interventions (for example, rotavirus due to difficulty controlling spread between young children) were excluded from the diarrheal disease mortality estimates shown in Table 3, providing a more realistic picture on how many deaths are considered preventable by WASH interventions.

Rising temperatures due to climate change are expected to exacerbate the burden of diarrheal disease. The WHO estimates an additional 48,000 deaths in children under 15 will be caused by climate change by 2030, and 33,000 by 2050. These estimates may be conservative since they do not account for diarrheal deaths caused by other risk factors such as declining water availability and undernutrition [39].

Cholera is an endemic diarrheal disease but is strongly associated with both natural disasters and civil conflict. There are an estimated 2.9 million cases of cholera causing 95,000 deaths each year in 69 endemic countries [40]. Cholera is transmitted through fecal contamination of water or food making clean water and sanitation critical to preventing its spread, but there is a lack of good evidence on which mix of interventions are most cost-effective during outbreaks (including oral cholera vaccine, case management and surveillance) as few high quality evaluation studies have been conducted [41].

Institutional settings such as schools, health facilities, prisons, and other public settings (refugee camps, public markets) can pose high risks if water and sanitation are not well managed. For example, studies have documented higher rates of diarrheal disease and gastrointestinal infection in schools lacking access to improved drinking water and sanitation facilities [42]. Improved hand hygiene is particularly important in institutional settings, given the ease with which infections spread in these environments.

##### Helminth Infections

Helminth infections are transmitted via fecal matter in water (schistosomiasis) and in soil (soil-transmitted helminths, STH). Although routine monitoring of infection rates is limited, a large number of prevalence surveys permits global estimates to be made. One study of helminth prevalence data for 6091 locations in 118 countries estimated that globally in 2010, 438.9 million people were infected with hookworm (*Ancylostoma duodenale*), 819.0 million with roundworm (*A. lumbricoides*), and 464.6 million with whipworm (*T. trichiura*) [43]. Of the 4.98 million years lived with disability (YLDs) attributable to STH, 65% were attributable to hookworm, 22% to *A. lumbricoides*, and 13% to *T. trichiura*. Most STH infections (67%) and YLDs (68%) occur in Asia (Central, East, South and Southeast). A separate study estimated 89.9 million STH infections in school-aged children in Sub-Saharan Africa [44]. Annual global deaths are estimated at 2700 for *A. lumbricoides* and 11,700 for schistosomiasis [45]. The global burden of schistosomiasis is estimated at 252 million cases in 2010, causing 11,700 deaths and leading to 3.1 million DALYs [46]. Three-quarters of this burden is suffered in sub-Sahara Africa.

Helminth infections cause several adverse health outcomes, including anemia, malnutrition, growth stunting, impaired physical and cognitive development, causing low school attendance and educational deficits, leading to loss of future economic productivity [47]. The risk of STH infection is greatest for specific occupations, such as agricultural workers, unplanned slums, poor populations, and those with poor sanitation and lack of clean water [48].

##### Undernutrition and Environmental Enteric Dysfunction

Undernutrition causes an estimated 45% of all child deaths [49] and is responsible for 11% of global disease burden [50]. Inadequate dietary intake and disease are directly responsible for undernutrition; however, multiple indirect determinants exacerbate these direct causes, including food insecurity, inadequate child care practices, low maternal education, poor access to health services, lack of access to clean water and sanitation, and poor hygiene practices [51]. Political, cultural, social, and economic factors play a role as well. Stunting (short height-for-age), underweight (low weight-for-age) and wasting (low weight-for-height) are all manifestations of undernutrition, which are associated with weakened immune systems. However, while a large proportion of children under five that die due to undernutrition are classified as both stunted and wasted, these conditions can have different determinants and respond to different interventions [50]. Wasting results from acute food shortage and/or disease and is significantly associated with mortality, while stunting results from chronic undernutrition, and is associated with severe long-term consequences, including poor cognitive development, lower school attendance, reduced human capital attainment, and a potentially higher risk of chronic disease in adulthood [47].

The links between diarrhea and child undernutrition [52,53] and other enteric infections [54,55,56,57] are well documented. An emerging body of evidence suggests that a subclinical condition of the small intestine caused by chronic ingestion of pathogenic microorganisms results in nutrient malabsorption and may be the primary causal pathway between poor water, sanitation and hygiene and child growth [58].

The evidence on the etiology of diarrheal disease finds an association between levels of intestinal inflammation detected through fecal samples and subsequent growth deficits in infants, lending support to the environmental enteropathy hypothesis that stunting may be an outcome of frequent enteric infection and intestinal inflammation [59]. Due to the asymptomatic nature of environmental enteropathy, the extent and seriousness of the condition is not known; however, it appears to be nearly universal among those living in impoverished conditions [60], and is hypothesized to be the cause of up to 43% of stunting [61].

The risks of low-birth weight and stunting are heightened in undernourished mothers [62], resulting in intergenerational consequences of undernutrition and related conditions.

#### 3.2.2. Impacts on Well-Being

Improved water supply and sanitation provide individuals with increased comfort, safety, dignity, status and convenience; along with broader impacts on the environment [63]. The social welfare impacts are difficult to quantify with certainty, given their subjective nature. Nevertheless, these benefits are consistently cited as among the most important for beneficiaries of water supply and sanitation [64,65], and may be particularly relevant for women [66].

Water supply within or adjacent to the housing compound provides greater comfort to household members, notably women and girls tasked with fetching water, while water sources closer to home, especially piped water, are associated with increased usage [67,68]. Data from 18 countries indicate that women are five times more likely than men to have the responsibility for collecting household water [12]. As the distance to the water source increases, the time that women could spend on income-generating activities, household chores, and childcare decreases [29]. A regular piped water supply can also open the possibility of purchasing time- and labor-saving devices, such as washing machines and dishwashers. While access to water infrastructure does not always translate into wage employment for women [69], one study found that it can reduce time spent on unpaid labor, improving gender equality [70].

On-plot sanitation reduces the risk of theft or assault (including rape and sexual harassment), especially at night or in isolated locations. Moreover, improved sanitation facilities are safer, less likely to collapse and easier for small children to use. Accompanying a child to the toilet is more convenient if it is nearby and safe, and mothers can comfortably step away from household duties to practice hygiene. In six countries of Southeast Asia, the time savings from rural households owning their own latrine varied from four to 20 min of travel time per trip [63]. In Ghana, more than 50% of households considering adopting a toilet included convenience in their top three reasons for investing in sanitation [71]. Privacy, comfort and convenience benefits are magnified for vulnerable groups, such as the elderly or persons living with disabilities or debilitating chronic illness.

Access to improved WASH services in schools and workplaces contributes to school attendance and performance and may influence decisions of where to work, especially for girls and women. Recent evidence from India shows that a national government program to build toilet facilities in schools led to an 8% increase in enrollment among pubescent-age boys and girls, and a 12% increase among younger children of both genders [72]. The comparably large effect of school sanitation on primary school aged children and robust effects for both boys and girls at all ages suggests that at least some of the impact of school sanitation is health related [42], and is not just a matter of privacy for pubescent age girls. Research has seldom analyzed academic performance as an outcome, but given the role that improved water and sanitation has on child health and attendance, this is a gap in the current evidence.

Menstrual hygiene management (MHM) is a poorly understood and under researched area of WASH services, whose neglect has left women in many low and middle-income countries without access to appropriate products, facilities, and services [73]. However, although lack of adequate MHM is frequently described as a hindrance to girls’ education, there is no high quality evidence to support this [74]. A randomized controlled trial in Nepal suggests that menses, and poor menstrual hygiene technology in particular, has no effect on absenteeism of girls; girls miss less than one school day per year on average due to menstruation [75]. However, girls may avoid going to school while they are menstruating not because of lack of management methods, but because they lack proper facilities for managing menses [42].

#### 3.2.3. Environmental Consequences

Two major environmental consequences of poor WASH practices are the excessive extraction of water to meet population needs and the pollution caused by poorly managed human excreta. While water supply for domestic use represents a small proportion of overall extraction, the concept of virtual water trade (the hidden flow of water if food or other commodities that require water to produce are traded from one place to another) has led to a greater understanding of the implications of population consumption patterns for water use. Globally, the combined effects of socioeconomic growth and climate change indicate that, by 2050, the population at risk of exposure to at least a moderate level of water stress could reach at least 5 billion people [76]. An estimated population of up to 3 billion in 2050 is nearly double the current estimate (~1.7 billion people) living under overly exploited water stress. Sadoff *et al.* present a risk metric of frequency of water shortage in reservoirs [77]. This metric is based on a combination of hydrological variability and water usage trends, which may in part be mitigated by storage infrastructure. This class of water insecurity is most severe in South Asia and Northern China, although the risk of water shortage exists in all continents.

Groundwater over-extraction and pollution of local surface water bodies has led many large urban population centers to source municipal water supplies from reservoirs or rivers that are tens or hundreds of kilometers from the site of treatment or consumption. These schemes cost tens of millions of dollars each in reservoir construction, pipeline, and pumping costs. Groundwater resources are increasingly under stress from unsustainable agricultural practices resulting from crop choice and energy subsidies to enable farmers to pump groundwater. In India and Mexico, for example, subsidized electricity and kerosene for farmers has led to serious groundwater overdraft [78].

Poorly managed human excreta has major environmental consequences, polluting human settlements, ground water, surface water such as lakes and rivers and eventually oceans. The degree of pollution is highly context-specific, depending on wastewater/sludge/sewage management practices, climatic factors, and the population size and density in relation to the volume of water. In highly populated river basins, municipal sewage and wastewater contribute a high proportion to overall biological oxygen demand [79,80]. Heavily polluted surface water has serious impacts on ecosystems, food webs, and biodiversity [81]. Coastal areas near the discharge of large, polluted rivers have reported compromised fish catch, such as in Argentina [82]. In coastal areas of the Philippines, water pollution was estimated to cost US$26 million per year in lost fish catch and degraded coral reefs [83]. Water pollution of recreational areas affects the tourism industry, either lowering visit rates or causing gastrointestinal illness.

#### 3.2.4. Financial and Economic Consequences

Financial and economic studies convert the health, social and environmental impacts of poor water supply, sanitation and hygiene to a common money metric, enabling aggregation as well as comparison across locations and over time. These estimates help policy makers, sector stakeholders and the general public understand the household-level as well as economy-wide consequences of poor WASH, and serve as the basis for cost-benefit analysis. However, these estimates are often incomplete, using crude estimates of economic value or relying on imprecise physical impacts underlying the economic values.

**Figure 4:**
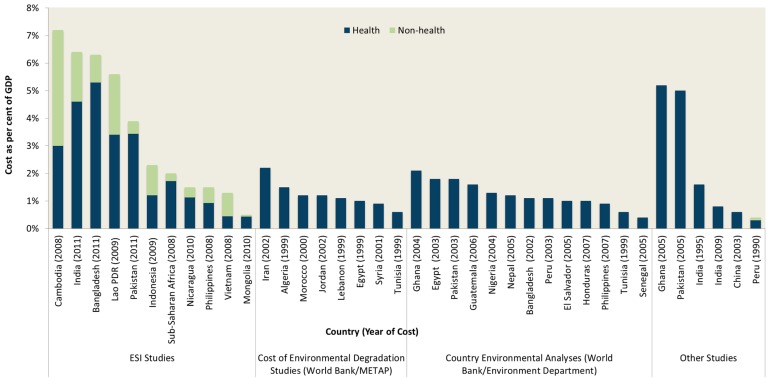
Economic costs of poor water and sanitation in selected countries, as a percent of gross domestic product, disaggregated by health and non-health damages. Source: Compiled by authors (see Table S1 for fuller data sets and references).

A global review of the economic consequences of poor water and sanitation (Figure 4 and Table S1) found the cost of poor sanitation exceeded 2% of total gross domestic product (GDP) in East Asian and Pacific and Sub-Saharan African economies, while in South Asia, it exceeded 4% of GDP. Although all the studies presented in Figure 4 present impacts in monetary units, the results are not directly comparable. They have different base years and different impacts included; some include only sanitation, and others include both water and sanitation. A global study, including the health and time losses, valued the costs in developing countries at 1.5% of global domestic product [84]. These significant economic impacts raise awareness as to the extent the problem, but they do not indicate how to address the problem in a cost-effective manner.

### 3.3. Effectiveness of Intervention Options

Three main categories of interventions to improve WASH are assessed:

- *Technology options and WASH practices* cover the type of hardware, equipment and associated behaviors of WASH services. Not all water or sanitation technologies perform the same function, so they can be classified by the service level they provide.
- *Service delivery models* cover the components of WASH service implementation. These include the approach to strengthening the supply chain, the approach to generating demand for WASH, the choice of implementing agency or WASH provider, and the extent of integration of WASH programs with other interventions.
- *Strengthening the enabling environment for WASH service delivery* includes measures to strengthen capacity, legal framework, policy and planning, resource allocation, monitoring and evaluation, and other interventions to provide a stronger foundation for implementing the technology and service delivery models.

#### 3.3.1. Effectiveness of Technologies and Practices

Water technologies are designed to source, treat, distribute, and monitor the supply of water. Epidemiological studies evaluate the effectiveness of water interventions in terms of the quantity and (microbial) quality of water supplied [85]. A growing evidence base enables the comparison of the incremental health benefits of different water interventions, between improved community source, piped water, higher quality piped water, and point-of-use treatment (chlorine, solar, and filter). However, there are insufficient studies to distinguish effects between the range of community water source interventions [86]. Utility regulators and regional/global initiatives monitor quality according to service standards, such as continuity, consumption, and number of complaints. In 2010, The International Benchmarking Network for Water and Sanitation Utilities (IBNET) of the World Bank reports that only 16% of utilities in low-income countries supply water continuously 24 h per day, compared to 86% of utilities in middle-income countries [87]. Even a few days of interrupted water supply can result in significant adverse health consequences if beneficiaries revert to using unimproved sources of water [88].

To increase safety, drinking water can be treated at the source or at the point of use through a process of filtration and/or disinfection. The largest health effects for improved water treatment technologies are for piped water supply, with a greater benefit associated with higher quality piped water (water that is safe and continuously available) [86]. Among household-level studies, filter interventions that also provided safe storage (for example, ceramic filters) were associated with a large reduction in diarrhoeal disease [86]. Neither chlorine treatment nor solar disinfection show significant impact on diarrhea after meta-analysis adjusted for non-blinding of the intervention [86], although an earlier systematic review and meta-analysis of water quality interventions found household level treatment was more effective than source treatment [88]. Blinding participants to the intervention and longer follow-up periods are recommended to better understand the impact of point-of-use water treatment interventions on diarrhea [89].

Sanitation technologies isolate, transport, and treat fecal waste to reduce the transmission of pathogens, and they provide users with a dignified and comfortable experience. Different rungs on the “sanitation ladder”, such as unimproved, improved on-site and improved with sewer connection, confer different health impacts [86] and user experience, and hence utilization can vary. Evidence shows that facilities shared by more than one household are associated with increased risk of diarrheal disease [90]. Public facilities are often poorly maintained and are less likely to be used by women, children and persons who are disabled or infirm. Distance also decreases usage of communal toilets [91].

Hygiene technologies enable users to perform basic personal hygiene functions. Epidemiological studies have typically used the presence of a place for handwashing with soap and water present as a proxy for handwashing practice; however this has been shown to be only loosely correlated with observed handwashing behavior [16]. A meta-analysis of hygiene interventions found an average risk ratio for diarrhea of 0.60 for promotion of handwashing with soap (95% CI: 0.53–0.68) and an average risk ratio of 0.76 in the risk of diarrhea for general hygiene education alone (95% CI: 0.67–0.86) (see Table 4) [15]. An earlier systematic review found a relative risk compared to no handwashing of 0.84 (0.79–0.89) for respiratory infection [22].

One synthetic review and meta-analysis of health impact assessments on diarrheal disease of water and sanitation interventions includes 61 individual studies for water, while for sanitation 12 observations compare unimproved with improved sanitation, and two observations compare unimproved sanitation with sewer connections [86]. Table 4 shows relative risk reductions for different movements up the water supply and sanitation ladders. The summary risk ratios of all observations on diarrhea morbidity is 0.66 (95% confidence interval: 0.60–0.71) for water interventions and 0.72 (95% Confidence Interval (CI): 0.59–0.88) for sanitation interventions [86]. An earlier review of 25 studies investigating the association between sewerage and diarrhea or related outcomes estimated an average risk ratio of 0.70 (95% CI: 0.61–0.79) increasing to as much as 0.40 when starting sanitation conditions are very poor [92].

The most recent meta-analysis of the impact of improved WASH on soil-transmitted helminths report overall odds ratios [93]. Access to sanitation was associated with decreased likelihood of infection with any STH (OR 0.66, 95% CI 0.57–0.76), *T. trichiura* (OR 0.61, 95% CI 0.50–0.74), and *A. lumbricoides* (OR 0.62, 95% CI 0.44–0.88), but not with hookworm infection (OR 0.80, 95% CI 0.61–1.06). Wearing shoes was associated with reduced odds of hookworm infection (OR 0.29, 95% CI 0.18–0.47) and infection with any STH (OR 0.30, 95% CI 0.11–0.83). Piped water access was associated with lower odds of *A. lumbricoides* (OR 0.40, 95% CI 0.39–0.41) and *T. trichiura* infection (OR 0.57, 95% CI 0.45–0.72), but not any STH infection (OR 0.93, 95% CI 0.28–3.11). Handwashing, both before eating (OR 0.38, 95% CI 0.26–0.55) and after defecating (OR 0.45, 95% CI 0.35–0.58), was associated with lower odds of *A. lumbricoides* infection. Soap use or availability was significantly associated with lower infection with any STH (OR 0.53, 95% CI 0.29–0.98), as was handwashing after defecation (OR 0.47, 95% CI 0.24–0.90).

Access to sanitation has been associated with lower trachoma as measured by the presence of trachomatous inflammation-follicular or trachomatous inflammation-intense with odds ratio 0.85 (95% CI: 0.75–0.95) and *C. trachomatis* infection with odds ratio 0.67 (95% CI: 0.55–0.78) [94].

A systematic review examined the impact of improved WASH on child nutritional status. Specifically, meta-analysis of five randomized controlled trials found a mean difference of 0.08 in height-for-age z-scores of children under age five years (95% CI: 0.00–0.16) for solar disinfection of water, provision of soap, and improvements in water quality [95]. However, the authors raised concerns about the low methodological quality of the included studies and the short follow-up periods; there was insufficient experimental evidence on water supply improvement and sanitation to include in the meta-analysis. Since publication of this review [95], several additional randomized controlled trials of household sanitation interventions have completed most failing to find a significant effect of the interventions in child health or growth outcomes. Two studies in India, one in Odisha [96] and another in Madhya Pradesh [97], found that while access to toilets increased in the intervention areas (51% in Odisha and 19% in Madhya Pradesh), open defecation practices continued, limiting the effectiveness of the intervention on child health and nutrition. A study of an earlier sanitation campaign in Maharashtra found modest improvements in village sanitation; but the study found these improvements were correlated with substantial increases in child height [98]. A similar study in Indonesia found no significant changes in access to improved toilets or open defecation practices, but detected a 30% reduction, on average, in diarrhea) [99]. In Tanzania, a sanitation campaign increased access to sanitation by 16% and reduced open defecation by 13%, but did not find effects on health [100]. One study of community-led total sanitation (CLTS) in rural Mali did lead to taller children on average (+0.18 height-for-age z-score, CI: 0.03–0.32), who were 6 percentage points less likely to be stunted after the intervention [27]. Econometric studies drawing on time series data establish links between open defecation, stunting [101]. 

The normative criteria of the Human Right to Drinking Water and Sanitation (acceptability, safety, availability, affordability and accessibility) encourage deeper assessments of how a technology or system is performing that go beyond the immediate health impacts, and were influential in defining the newly adopted WASH targets, indicators and definitions in the SDGs [2].

A source of regularly updated evidence reviews on WASH interventions with strict inclusion criteria is the Cochrane Library [102]. Other assessments are provided elsewhere, considering affordability [103], economic impacts (Table S1) and intervention efficiency (Tables S2 and S3).

#### 3.3.2. Effectiveness of Service Delivery Models

Effectiveness of service delivery models is measured in terms of intervention uptake, change in risky behaviors, sustainability, and to a lesser extent, health outcomes. Large-scale approaches that include demand raising and behavior change are needed to achieve universal access, but experience has shown these result in lower average effectiveness. A variety of financing approaches have been used to stimulate the market for WASH services, including private sector participation and grant financing, with subsidies being provided to both suppliers to strengthen service delivery and to households to stimulate demand for services.

Specific behaviors, such as household water treatment and storage (HWTS) and handwashing with soap (HWWS), have been the subject of behavior change campaigns. Despite substantial evidence pointing to health benefits of HWTS, skepticism remains that the results may largely be due to bias and concerns remain about the extent of uptake, use, and scalability of commercially marketed HWTS, particularly among poor populations most at risk of diarrheal disease [104]. Similarly, there is limited experimental evidence of the impacts of handwashing behavior change interventions [105,106]. Public Private Partnerships for Handwashing (PPPHWs) combine the marketing expertise of the soap industry with government support and enabling environment to trigger behavior change. Evaluations of PPPHWs have been commissioned by private soap companies and involved free provision of soap to households [107], thus limiting their external validity.

Demand-based approaches are now implemented in well over 50 developing countries where there have been many thousand applications of sanitation promotion and approaches such as Community-led total sanitation (CLTS). At least 16 national governments have adopted CLTS as national policy. These approaches start from the premise that real and lasting change is brought about when individual and community behaviors are impacted. One of the few rigorous evaluations of the CLTS approach comes from a recent example in rural Mali, in which CLTS was well-implemented to a random set of villages and shown to almost double coverage of a private latrine [27].

Supply-side approaches to water and sanitation service delivery cover the full value chain from production and assembly of inputs, importation, sales, distribution, installation, and maintenance of water infrastructure and latrines. These services range from micro- and small-scale independent water resellers, network operators, well and pit diggers, and operators offering masonry, pit, and septic tank emptying, and public toilet operators, to medium scale sanitation markets or “sanimarts” offering a full range of sanitation goods and services. Small-scale operators can effectively serve rural markets where the majority of people without access to piped water and sanitation live, but the existing literature highlights several obstacles to growth and the ability of these providers to effectively serve these populations [108].

Supply-side strengthening is predominant in the Community Approach to Total Sanitation (CATS) promoted by UNICEF [109] and the Total Sanitation and Sanitation Marketing (TSSM) approach developed by the World Bank Water and Sanitation Program [110]. Recent randomized control trial impact evaluations of TSSM in Madhya Pradesh, India (which included a hardware subsidy to below poverty line households), East Java, Indonesia, and 10 rural districts of Tanzania found the approach varied widely in its effectiveness across the countries with no increase in improved sanitation in Indonesia [99] and increases of 19% and 15.7% in Madhya Pradesh [111] and Tanzania [112], respectively. Despite better sanitation coverage in Madhya Pradesh large numbers of adults continued to practice open defecation. Another recent randomized intervention study in Bangladesh compared the effectiveness of a supply-side market intervention (sanitation extension agents) against community sanitation promotion (CLTS approach) and subsidies [113]. Neither community promotion nor the supply-side intervention led to a measurable increase in coverage when delivered in isolation. However, households who received subsidies alongside community promotion increased latrine adoption by 22%. Open defecation also declined, but not by the same order of magnitude (14%).

Results-based approaches (RBA) that offer financial or non-monetary rewards upon demonstration of measurable outputs or outcomes are increasingly part of the mainstream for achieving desirable outcomes in development sectors [114]. The specific details differ, but these approaches share a common aim of shifting the overall incentive structure from financing infrastructure to delivering services. Until recently, the experience using RBA in water and sanitation was limited. A review by the World Bank in 2010 indicated that less than 5% of its output-based-aid (OBA) portfolio was in water and sanitation [115]. The use of OBA has since increased under the Global Program for Output Based Aid (GPOBA), which lists 22 projects in water supply and sanitation whose outputs include water, sewerage, or sanitation connections [116]. Multilateral and bilateral agencies such as the World Bank, Inter-American Development Bank and Department for International Development (DfID) have shifted funding towards RBA in water and sanitation. The World Bank’s relatively new instrument Program for Results Based Financing (PforR) has several active operations in water supply, sanitation and hygiene [117].

Consumer financing can help poor households facing liquidity constraints to invest in water supply and sanitation by smoothing consumption over time, make them more willing to adopt improved services and giving them an opportunity to purchase more durable, higher levels of service. Consumer credit has been applied successfully to increase the take-up of household piped water connections [118], but experimental evidence of consumer lending for sanitation remains limited. However, emerging interest in the potential of consumer lending for household sanitation and results of small-scale pilots are promising. For example, a randomized study in Cambodia found a four-fold increase in uptake when households were offered a 12 month low-interest loan to purchase a latrine [119].

#### 3.3.3. Effectiveness of the Enabling Environment

In the face of slow progress in WASH coverage in many developing countries, and remaining problems in sustaining WASH services, the “enabling environment” has gained greater attention in the past decade. The enabling environment has been variously defined and classified. The World Bank has defined eight enabling environment dimensions considered essential to scaling up rural sanitation, comparing performance over time in three pilot countries (India, Tanzania and Ethiopia): (1) policy, strategy, and direction; (2) institutional arrangements; (3) program methodology; (4) implementation capacity; (5) availability of products and services; (6) financing and incentives; (7) cost-effective implementation; (8) monitoring and evaluation [120]. An assessment of progress between the 2007 baseline and the 2010 endline strongly suggests that the countries with the strongest enabling environment made the most progress in sanitation coverage [120].

The Country Status Overview (CSO) is a methodology that has been applied in 32 African countries in 2010—adopted three service delivery cycles, each containing three building blocks: (1) enabling: policy, planning and budgeting; (2) developing: expenditure, equity and service outputs; and (3) sustaining: maintenance, expansion and use [121]. Since then, similar assessments (where they are called “Service Delivery Assessments”—SDA) have been applied East Asian and the Pacific [122] and in South Asia [123].

An initiative that has built on the above frameworks is called the WASH Bottleneck Analysis Tool (WASH-BAT) [124]. The tool enables detailed, separate assessments of rural water, rural sanitation, urban water and urban sanitation, at various levels—national, sub-national, service provider and community. The aim of the tool is that it is applied jointly in a meeting of sector stakeholders, which increases transparency, objectivity and buy-in, and encourages government leadership.

At global level, separate assessments have been conducted under the UN-Water Global analysis and assessment of sanitation and drinking-water (GLAAS), which covers 74 developing countries and 24 external support agencies and includes indicators on policies, planning, coordination, financing, human resources, equity and external support [125]. The GLAAS report is used as an advocacy tool to bring greater attention to key drivers of sector progress, and is reported at the biennial “High Level Meetings” organized by the Sanitation and Water for All (SWA) partnership.

Together, these initiatives have covered the majority of developing countries. They have successfully highlighted the importance of supportive policies, transparency and collective action in building a strong sustainable sector. With the experiences of these various initiatives comes important learning on the relative importance of different levers to advance the WASH sector.

### 3.4. Intervention Costs, Benefits, Efficiency, and Sustainability

As societies do not have limitless resources, any intervention in the WASH sector requires an economic rationale, thus satisfying conditions of efficiency, affordability and relevance (*i.e.*, meeting a need or demand). *Cost-benefit analysis* compares the intervention costs with the benefits, expressed in monetary units. *Cost-effectiveness analysis* compares the intervention costs with the benefits, expressed in terms of some other common unit, such as lives saved or pollution load to the environment averted.

#### 3.4.1. Costs

The cost of interventions is one key piece of evidence for decision making, because it is relatively easy to obtain and is an often-cited constraint for an investment decision, whether governments, the private sector, or households and individuals. Costs can be measured for the WASH technology (the hardware), the service delivery approach (the “software” or program management), and the enabling environment.

Despite its importance, cost information is not commonly tabulated in appropriate format to support decision making. At the policy level, budgets and resource allocations are fragmented among subsectors, levels of government, and sector partners or financiers. Considerable differences exist between budget allocations and disbursements. The WASH-BAT is a tool developed by UNICEF that helps to consolidate the budgetary needs for removing major sector bottlenecks (see Section 3.3.3) [124]. At the program or service delivery level, implementers do not easily share information on their costs, and budgets may not be structured to provide simple break down between software and hardware costs. For WASH technologies, the cost studies are more abundant, and at local level the market or subsidized price is available. However, the price is rarely exactly the same as the cost, as the price commonly contains either a profit or a subsidy, and as both are transfer payments they should ideally be excluded from economic analysis. However, to ease the research burden it is common practice for economic analysis to use prices as a proxy for cost, adjusting for any known subsidy or profit.

**Figure 5:**
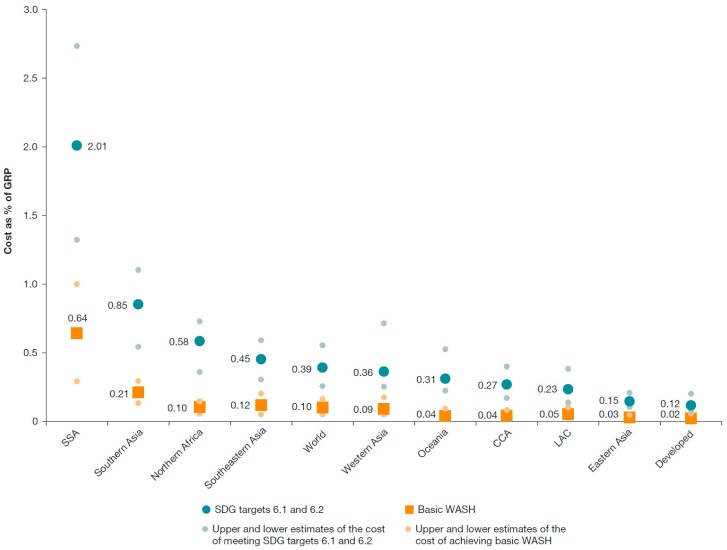
Costs of basic and safely managed services as percentage of gross regional product (GRP) by MDG region, with uncertainty range. Reproduced with permission from the World Bank [126]. Note: WASH = water, sanitation, and hygiene; SDG = Sustainable Development Goal; SSA = Sub-Saharan Africa; LAC = Latin America and the Caribbean; CCA = Caucasus and Central Asia. Gross regional product is based on the aggregated GDP of countries in each region. An economic growth rate of 5 percent is assumed all regions. Lower and upper bounds were based on three significant sources of uncertainty: (1) 100 percent of population using low-cost technology to 100 percent using high cost technology (baseline 50% each); (2) discount rate varied from 3 percent to 8 percent (baseline 5%); and (3) alternative method of transferring cost data to countries with limited unit cost data, using absolute U.S. dollar values instead of adjusting taking into account differences in purchasing power.

Published cost evidence is available in both aggregated and unit form. Aggregated cost includes the expenditure required to meet specified targets. For example, the World Bank estimates the global capital costs of achieving universal access to WASH services by 2030 are US$ 28.4 billion per year (range: US$13.8 to US$46.7 billion) from 2015 to 2030 for basic WASH (similar to “improved” standard), and $114 billion per year (range: $74 to $166 billion) for safely managed WASH [126]. These costs are equivalent to 0.10% of global product for basic WASH and 0.39% of global product for safely managed WASH, including the 140 developing countries [126]. These needs compare with 0.12% of its product spent on meeting the MDG water and sanitation target: hence universal basic access by 2030 is potentially feasible at current spending but requires reallocations to sanitation, to rural areas and to off-track regions [126]. However, substantial further spending is needed to meet the higher standard of safely managed services. The costs as a proportion of gross regional product are shown by MDG region in Figure 5. Regions most challenged to reach universal access are sub-Saharan Africa and Southern Asia. Many countries also produce investment plans for meeting national targets, focusing on the financing to be provided by government. The Organisation for Economic Co-Operation and Development (OECD) has created a tool called FEASIBLE for developing national financing strategies by comparing the costs of meeting national targets with projected financing available [127]. The tool has been applied in at least 12 countries [128].

A key input to these aggregated studies is the unit costs of WASH provision at the household or community level. Due to climatic, topographical and socio-economic differences, costs of service provision are highly variable between studies, contexts and levels of service. The cost per m^3^ of water and wastewater services, as well as average monthly household bills, are available for utility services via national regulators, as well as regional associations and global initiatives [87]. Studies commonly compare the cost of different sources of water supply, finding piped water to be significantly cheaper on a per unit basis compared with vendor supplied water, while monthly expenditure is more similar due to higher consumption of piped water than other water sources [129]. On the other hand, cost per household served is usually significantly less for community water interventions (e.g., borehole, tubewell) than for household piped water [126]. The WASHCost project calculated benchmark capital and recurrent costs for basic levels of water service in Andhra Pradesh, India, Burkina Faso, Ghana and Mozambique [130]. Benchmark capital costs ranged from US$20 per person for boreholes and hand pumps to US$152 for larger water schemes. Benchmark recurrent costs ranged from US$3 to US$15 per person per year, but actual expenditures were substantially lower. Construction cost per household for sanitation varies widely between settings, for example in rural areas a dry pit latrine varied from US$37 in the Philippines to US$158 in Cambodia, and in urban areas sewerage varied from US$513 in Indonesia to US$5345 in Cambodia [63]. Comparison of alternative sanitation transportation and treatment technologies also provides important policy direction—in general fecal sludge management is considerably cheaper than sewerage such as in Dakar, Senegal, where it was found to be five times cheaper [131].

Ideally, the costs of water supply and sanitation services should consider the externalities and the long-run cost of supply. One study provides an illustrative example of the full costs of water supply and sanitation (including opportunity costs and environmental costs) with the low costs, varying from a high of US$2.00 per m^3^ to a low of US$0.80 per m^3^, shown in Table 5 [129].

From a policy perspective, the affordability and willingness to pay for these costs is a critical issue. A global review found that water supply costs as a proportion of household income is significantly higher for poorer populations [103], well above the benchmark of between 3% and 5% used by some governments and international organizations.

#### 3.4.2. Benefits

WASH services have a large array of welfare and development benefits. Table 6 classifies these benefits under health, convenience, social, educational, reuse, water access, and other benefits.

These benefits have been evaluated extensively, but few studies evaluate benefits comprehensively. The most robust scientific studies, such as randomized or matched prospective cohort studies, have been conducted on health impacts, but there are only few of these, and economic variables are rarely captured. The majority of economic studies build models filled with input data from a mixture of sources. Global studies assessing the economic benefits of improved water supply and sanitation include health economic and convenience time savings [84,132]. Country studies have also evaluated the value of health and time savings [133]. Regional studies from Southeast Asia assess the water access, reuse, and tourism benefits of improved sanitation [63,134] as a proportion of avoided damage costs (Figure 4).

Willingness to pay studies have estimated economic value of water quality improvements [135,136], while others have assessed willingness to pay to avoid health impacts [137,138] and to receive piped water [139]. A systematic review has shown that willingness to pay for water quality improvement is less than the cost of producing and distributing it [139]. Social benefits have been assessed, but few have expressed in money values, except willingness to pay studies, which tend to capture all benefits and make difficult to differentiate social from other benefits.

Economic value is associated with river clean up that includes improved management of municipal wastewater as well as improved management of industrial discharge, agricultural runoff, and solid waste [140]. The financial viability of WASH services is expressed in terms of financial returns. The most comprehensive source of data is from projects of multilateral development banks that routinely conduct a financial assessment of WASH services prior to project approval, and in some cases, provide a project implementation completion report.

#### 3.4.3. Intervention Efficiency: Cost-Benefit Analysis

The discussion of efficiency should distinguish between cost-benefit analysis, which uses a common money metric for all costs and benefits, and cost-effectiveness analysis, which compare interventions for one type of outcome. Reviewed cost-benefit studies are provided in Table S2.

Efficiency studies can be conducted in two ways [129].

- Generate estimates of cost and benefit in specific sites or field studies, for the purposes of either evaluating intervention performance or selecting a site for a future project [63].
- Model costs and benefits for specific sites or larger jurisdictions, such as country or global level, using best-available evidence from multiple sources [129,132].

Given the high costs and challenges associated with collecting all the cost and benefit data required for the first approach, it is common practice to combine site-specific values with data extrapolated from other sources [63]. Table 7 shows the latest available global studies that have modeled selected water supply and sanitation interventions. One important finding from these studies is that lower technology interventions have higher returns than more expensive networked options.

Global studies indicate the projected overall costs and benefits from intervention alternatives, but they are not particularly useful in guiding decisions on which technology and service level to choose in specific settings. In a review of willingness-to-pay (WTP) studies for improved water supply in low- and middle-income countries, 40 studies provided 137 estimates of WTP [135]. The authors compared average WTP with costs of service provision in three regions; they found that WTP exceeds costs for improved water coverage, while costs exceeded WTP for piped water coverage. One randomized implementation study in India finds similar health costs between study arms but a statistically significant reduction in time savings in the intervention group of US$7 per household (US$5 for water and US$2 for sanitation) during the dry season, or roughly 5% of monthly cash expenditures [141]. A study from South Africa estimates a benefit-cost ratio of 3.1 for small-scale water schemes [142]. A study from Indonesia compared three wastewater treatment interventions and finds limited economic rationale for the interventions [143]. On the other hand, a broader cost-benefit study at the river basin level estimated the benefits of cleaning up the Upper Citarum River in Indonesia exceeded costs by 2.3 times [140].

Targeting the poor could be justified by the fact that children from poorer households are at increased health risk as they live in communities with lower access to improved water and sanitation facilities. A study in Bangladesh, India, and Pakistan estimating cost-per-episode for income quintiles shows that costs of an illness represent a higher proportion of income for lower quintiles [144].

The cost-efficiency of technologies is context specific and depends on the local geological setting, population density, and number of households to be served. For example, large water distribution and sewerage systems may only be cost-efficient if serving large, dense populations; smaller-scale water service provision via either communal or in-compound wells or boreholes and onsite household sanitation may be a more appropriate and cost-efficient service level for sparsely populated areas [145].

#### 3.4.4. Intervention Efficiency: Cost-Effectiveness Analysis

The main outcomes used in cost-effectiveness studies are health and environment related. To compare programs within a sector, cost-effectiveness can be measured in terms of programmatic outcomes such as number of latrines constructed, water connections installed, or percentage of beneficiaries changing behavior. For water supply interventions, a number of health cost-effectiveness studies have been conducted (see Table S3). Studies focus on improved water supply (as per JMP definition) and point-of-use treatment by households or schools. A global study compares water supply interventions at the regional level [90]. It should be noted that cost-effectiveness analysis (CEA) focuses on measurable health outcomes but exclude user preferences are a major determining factor in technology choice.

**Figure 6:**
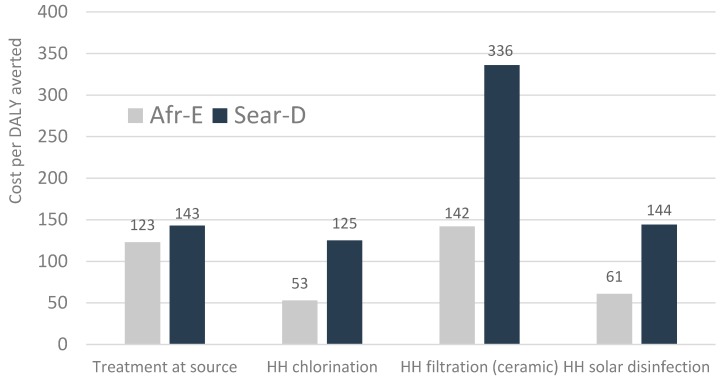
Cost per disability-adjusted life-year (DALY) averted from treatment at source and four household (HH) water supply and water quality interventions in two WHO sub-regions. Africa Epidemiological Stratum D and South and SE Asia Epidemiological Stratum D (US$, 2005). Source: [89].

Figure 6 shows the cost per healthy life year gained for four interventions in two regions, showing that the selected interventions vary by a factor of approximately 2.5 between the most cost-effective (chlorination) and the least cost-effective (ceramic filter). However, all interventions have a cost per healthy life-year (HLY) that is below the GDP of countries in these regions, indicating a cost-effective use of health resources. Another global study found the incremental costs averted of adding point-of-use water disinfection on top of improved water supply costs resulted in cost per disability-adjusted life-year (DALY) averted of less than US$25 in Sub-Saharan Africa, US$63 in India and Bangladesh, and less than US$210 in Southeast Asia and the Western Pacific regions [146]. 

Health cost-effectiveness analyses of sanitation and hygiene interventions have been conducted in fewer studies. Two global studies by the WHO and World Bank examine cost-effectiveness of water supply and sanitation combined [146,147]. Using regions defined by epidemiological strata, the WHO estimates the cost in countries with high child and high adult mortality is less than US$530 per DALY averted in Eastern Mediterranean and Middle East, US$650 in Africa, US$1400 in South and Southeast Asia, and US$2800 in Latin America and the Caribbean. A World Bank study, focusing on child mortality reductions, estimates the average cost per life year saved in Sub-Sahara Africa countries of US$1104 for basic improved water and sanitation and US$995 for privately piped water and flush toilets [147].

In country studies in Southeast Asia, the cost per DALY averted of basic sanitation is less than US$1100 in selected rural areas of Cambodia, China, Indonesia, Lao PDR, and Vietnam; the exception is in the Philippines, where it is US$2500 [63]. Few recent country-specific studies are available on hygiene interventions; one study from Burkina Faso estimates a cost of US$51 per death averted for health education to mothers [148].

Environmental cost-effectiveness studies compare the costs of achieving pollution or nutrient emission reductions through different approaches to wastewater or fecal sludge management [149,150]. The majority of studies have been conducted in developed countries.

#### 3.4.5. Sustainability

WASH intervention sustainability can be examined from several angles—whether interventions are functionally sustained (*i.e.*, maintained), environmentally sustainable (water extraction at renewable rates, and energy use in WASH service provision and thus greenhouse gas emissions), and financially sustainable.

The challenge of any service is that after the initial investment, the proportion of population using the service declines over time due to a variety of reasons that include both supply (e.g., hardware breakdown and lack of replacement or maintenance) [151] and demand issues (e.g., lack of sustained demand for the services often due to a preference of populations to meet their WASH needs other ways) [152]. Sustainability is a challenge for both WASH behaviors such as handwashing with soap, as well as WASH infrastructure. Increasingly, evidence has become available on the extent to which services are not sustained [153]. However, there are few rigorous studies which capture a meaningful time frame to measure sustainability of outcomes. For sustainability of rural water supply, research in Liberia, Sierra Leone, Uganda shows that while quality of construction plays a role, poor management and lack of operations and maintenance are the primary drivers [154]. The bulk of the evidence suggests that handwashing with soap behaviors are not sustained long after the intervention [155]. Funding and partner agencies have increased their focus on service sustainability leading to the development of new sustainability indicators [156].

The energy-water nexus is now coming to the fore, raising the issue not only of the energy requirements of water supply and wastewater systems (including transport, treatment and disposal), but also the resulting over extraction of groundwater resulting from energy subsidies and polluted surface water. In India and Mexico, for example, subsidized electricity and kerosene for farmers has led to serious groundwater overdraft [78]. Municipal water supply is also being sourced from further away in several mega-cities (e.g., Beijing, Metro Manila, Dhaka, Mexico City) due to declining water tables and polluted local surface water such as natural lakes and rivers, thus costing tens of billions of dollars in reservoir, pipeline and/or pumping costs.

Wastewater transportation and treatment require considerable amounts of energy. Evaluations of alternative wastewater treatment systems show that wetland systems can use as little as 15% of the purchased energy of conventional sewage systems [157]. Furthermore, the systems vary in terms of their greenhouse gas emission [158]. Emissions can be cost-effectively reduced by capturing methane emission and using it as a source of energy for the rest of the treatment process [159].

The main sources of financing are the three ‘T’s—transfers, taxes and tariffs [160]. These sources not only need to expand coverage to meet global and national targets, but also need to maintain existing coverage, including maintenance, rehabilitation and where necessary, replacement. While the estimates of global investment needs for water supply and sanitation are available (e.g., see Table 1), the global estimates of current financing are unavailable [125]. First, it is largely unknown how much households are spending. Second, few developing country governments routinely provide breakdown between different line items to enable separation of water and sanitation in budgets and expenditure at both central and decentralized levels [125]. Third, overall official development assistance (ODA) amounted to US$ 10.9 billion in 2012, or 6.1% of reported ODA [161]. Some donors are increasingly reporting annual disaggregated disbursement on water and sanitation projects, but many transfers are excluded such as from non-governmental organizations. A decade ago, a landmark report from the Report of the World Panel on Financing Water Infrastructure chaired by Michael Camdessus ventured that current financing needs to double to meet the MDG targets for water supply and sanitation [162]. Given how far short the world fell in meeting the sanitation MDG target, financing clearly did not keep up with needs. A more recent paper from the World Bank proposes four main ways of making up the financing deficit: more efficient operations of service providers, increase tariffs towards full cost recovery, more public resources allocated, and government and donors leveraging investments from municipal bonds and the private sector [163]. Furthermore, spending should be directed towards poor people and rural areas of the poorest countries with the greatest WASH challenges. A long-term vision with a solid strategy based on solid data is considered as key for moving forward [164]. The OECD has defined a methodology and software tool called FEASIBLE that has been implemented in many countries [127].

The ability to mobilize financing will be critical in achieving universal access of safely managed WASH services by the year 2030 [126]. However, given the variable performance of utilities [87] and poor budget absorption [125] in many low- and middle-income countries, the ability to translate financing into effective services will be even more critical. Furthermore, given the insufficiency of public funds to meet the targets, these will need to be targeted at households less able to afford WASH services [103]. Benchmarks for proportion of household income (or expenditure) on water and sanitation services vary between 3% and 5%—however, the ability to capture the full costs of WASH services is limited from existing household surveys or other data sources [165]. A benchmark for government expenditure was proposed at 1% of GDP on water supply and sanitation capital expenditures [166]. However, current public spending in a sample of 15 African countries is 0.32% of GDP [167].

## 4. Discussion

Since reliable global data sets have been available (and comparable over time) during the MDG era, important progress has been made towards global water and sanitation targets. At current rates of progress and using current indicators, achieving universal access targets will take at least 20 years for water supply and 60 years for sanitation [18]. Covering the poor and marginalized populations will continue to be a challenge for some time, as the remaining unserved populations are likely to be harder to reach as universal access is approached. However, in September 2015 the UN General Assembly endorsed a new set of global targets under the SDGs. The service level benchmark aspired to—“safely managed” services—goes beyond the “improved” definition of the MDG period. This raises a new set of challenges, on the global monitoring of new definitions and indicators and on the set of policy, regulatory and spending requirements to achieve a higher WASH standards. The new standards will raise questions about priorities, and countries will face a trade-off between dedicating policy space and public subsidies on moving already-served populations higher up the water and sanitation ladders *versus* reaching the unserved with basic WASH services. While each country will have its own unique set of challenges to deal with, the human right to drinking-water and sanitation should provide the reminder that priority should be given to ensuring at least a minimum level of affordable WASH service for all the world’s citizens.

The global WASH sector is not static, neither are populations or the economic context. Populations are growing and moving, economies are developing and becoming richer, and the climate is changing. Each one has its challenges and opportunities. Population migration to greenfield sites offers a chance of implementing new and appropriate technologies, ensuring these not only meet the expectations of populations but are also cost-effectively and affordably implemented. Economic growth also leads to greater tax revenues of local governments and enhances their ability to upgrade infrastructure and expand urban renewal. Climate change challenges the delivery of WASH services by affecting rainfall patterns, freshwater availability, and frequency of heat events. At least 2.8 billion people in 48 countries will be affected by water stress by 2025. However, this new threat, when taken seriously, can be an opportunity to overhaul outdated policies and technologies. Furthermore, as nutrient sources for chemical fertilizer become scarcer, price increases will force suppliers to seek alternatives; the price of composted sludge is expected to increase, attracting investments. While climatic factors are harder to control, water scarcity can be mitigated by changing water usage patterns and reducing pollution of surface waters. New research, data and technologies are becoming available at an increasing rate, thus opening new possibilities for dealing with seemingly entrenched problems in the WASH sector.

On the health front, while global deaths from diarrhea have declined significantly over the past 20 years, poor water supply, sanitation and hygiene are still responsible for a significant disease burden. An estimated 842,000 global deaths were due to diarrhea caused by poor WASH in 2012, and there remain other less well quantified but important long-term health impacts of poor WASH, such as helminthes and enteric dysfunction. These diseases affect children’s nutritional status, inhibiting growth and mental development. WASH-related epidemics—whether regular ones such as cholera or ones that mobilize global responses such as Ebola—affect the poorest most of all, and can devastate communities. Overall, the health impacts of poor WASH lead to economic consequences in the order of several percent of GDP, even in large middle-income countries, and continue to significantly affect people’s quality of life and the environment.

To adequately address equity considerations in the post-MDG era, there is a need to understand where the poor live and what their levels of access are. Disaggregated data on the underserved—including slum populations, ethnic groups, women, elderly, and persons with disabilities can also support prioritization. Greater focus is needed on how to increase access in the lagging regions of South Asia and Africa where a large proportion of the unserved live. At the country level, policy and financial incentives need to be aligned and the economic arguments made for allocating resources to WASH services, especially to sanitation. National financing strategies that engage a fuller range of stakeholders, including the private sector and non-traditional financing sources, will expand the resources drawn into the provision of WASH services; these strategies also need to be translated to lower administrative levels.

More evidence is needed to support our emerging understanding of the wider health effects of water, sanitation and hygiene. The social welfare consequences of poor WASH are not well documented, but are potentially very large. In particular, a greater understanding of the gender impacts of inadequate WASH and how improved WASH services contribute to gender equality is needed. The role of multi-sectoral approaches will become more important as the complementarities between WASH, health and nutrition are better understood. Further rigorously designed, controlled studies are needed to quantify these benefits, including measurement of cost-effectiveness to guide policy and program design.

A large part of the remaining challenge of improving access to sanitation and hygiene is behavioral rather than technical but there is little evidence that behavior change using conventional methods is effective at scale, or that behavior change interventions that are successful in a particular context are effective elsewhere. A better understanding of habit formation and what leads to sustainable behavior change is needed. Given the continuing rate of rural-urban migration, a better understanding is needed on which WASH interventions work in slum areas and low-income neighborhoods, and under what conditions they work.

Innovative delivery platforms that leverage national poverty reduction programs, such as conditional cash transfers (CCT) and community driven development (CDD) programs have potential to achieve wide coverage at little marginal cost. These approaches can also provide the methodology and data sources to support poverty targeting of WASH services. There is also a need to understand how output-based approaches can be used to improve WASH service delivery and lead to greater sustainability of services. Innovations in subsidies and consumer financing have been shown to help the poor gain access to improved sanitation.

## 5. Conclusions

This review has shown there exists significant evidence on many aspects of WASH which can be utilized in designing and implementing improved policies and programmes. Over time, global data sets on WASH coverage are improving and better quality research is available on the impacts of inadequate WASH and the effectiveness of WASH interventions. However, to optimize available resources, further evidence is still needed. This relates in part to the expanded scope of global targets—higher service levels, the inclusion of hygiene and the recognized need for better institutional WASH. It also relates to the need to achieve better targeting of programmes to poor and marginalized households and communities (including children and women), improved mechanisms for achieving demand creation and behavior change, and some of the challenges we face on continued urbanization, population growth and climate change. While global overviews of evidence are useful as a first step, to be truly useful for WASH decision makers—evidence needs to be compiled and reviewed that relates to specific contexts, such as rural or urban areas, or at country or regional level.

## Figures and Tables

**Table 1:** Scope of water, sanitation, and hygiene services included.

Service	Included	Excluded
Water supply	Water for drinking; Other water uses in the home (cooking, hygiene, sanitation, cleaning, laundry); Treatment, safe handling and storage of water	Water for productive uses
Sanitation	Toilets and onsite excreta management; Management of fecal sludge; Sewerage or combined sewer-drainage systems	Separate gray water management; Industrial wastewater management; Storm water drainage; Solid waste management
Hygiene	Hand washing; Menstrual hygiene management	Food hygiene; Environmental hygiene and cleanliness measures; Other personal hygiene practices, including face and body cleansing

Source: Authors.

**Table 2:** Definitions of service levels proposed for monitoring of the WASH-related targets of the Water SDG #6. The higher level service indicators are proposed for SDG monitoring.

Service	First Service Level (Termed “Basic WASH”)	Higher Level Service (Termed “Safe WatSan”)
**Water**	Percentage of population using a protected community source or piped water ^1^ with a total collection time of 30 min or less for a roundtrip including queuing (termed “basic” water)	Percentage of population using safely managed drinking water services. “Safely managed” refers to an improved^1^ drinking water source on premises accessible to all members of the household, which delivers sufficient water to meet domestic needs, was functional >12 days in the last 2 weeks, meets WHO guideline values for *E. coli*, fluoride and arsenic, and is subject to a verified risk management plan [3].
**Sanitation and hygiene**	Percentage of population not practicing open defecation. Percentage of population using a basic, private sanitation facility (termed “adequate” sanitation) ^2^	Percentage of population using safely managed sanitation services including a hand washing facility with soap and water. “Safely managed” refers to safe capture of fecal waste with isolation or treatment with safe disposal/reuse, either on or off site. When off-site, fecal waste is safely extracted and conveyed to treatment and disposal sites.
	Percentage of population with handwashing facilities with soap and water at home.	

Source: Definitions of “improved” [4]; definitions of new indicators [2]. ^1^ Same as “improved“ water monitored as part of the MDG Target 7c (*i.e.*, piped water into dwelling, plot, or yard; public tap/standpipe; tubewell/borehole; protected dug well; protected spring; rainwater collection); ^2^ Same as “improved’” sanitation monitored as part of the MDG Target 7c (*i.e.*, flush or pour-flush to piped sewer system, septic tank, pit latrine or ventilated Improved Pit-latrine; pit latrine with slab and composting toilet).

**Table 3:** Diarrheal Disease Mortality Attributed To Poor Water Supply, Sanitation, and Hygiene in Low-and Middle-Income Countries, Regional and Risk Factor Breakdown.

Region	Water Supply	Sanitation	Hygiene	WASH
Africa	229,316	126,294	122,955	367,605
America	6441	2370	5026	11,519
Eastern Mediterranean	50,409	24,441	28,699	81,064
Europe	1676	352	1972	3564
South & Southeast Asia	207,773	123,279	131,519	363,904
Western Pacific	6448	3709	6690	14,160
World	502,061	280,443	296,860	841,818

Source: [36]. WHO Regional classifications. Totals may not be sum of rows due to rounding. Columns 2–4 do not sum to column 5 due to overlap in risk pathways.

**Table 4:** Meta-regression results for water and sanitation interventions: relative risks of diarrhea compared with no improved water, sanitation, or hygiene practice (95% confidence intervals in brackets).

Baseline	Intervention			
Baseline water	Improved community source	Piped water, non-continuous	Piped water, high quality	Filter and safe storage in the household
Unimproved source	0.89 (0.78, 1.01)	0.77 (0.64, 0.92)	0.19 (0.07, 0.50)	0.53 (0.41, 0.67)
Improved community source		0.86 (0.72, 1.03)	0.21 (0.08, 0.56)	0.59 (0.49, 0.78)
Basic piped water			0.57 (0.09, 0.65)	0.69 (0.51, 0.93)
Baseline sanitation	Improved sanitation, no sewer		Sewer connection	
Unimproved sanitation	0.84 (0.77, 0.91)		0.31 (0.27, 0.36)	
Improved sanitation, no sewer			0.37 (0.31, 0.44)	
Baseline hygiene	General hygiene education		Handwashing with soap	
No hygiene education or handwashing	0.76 (0.67, 0.86)		0.60 (0.53, 0.68)	

Sources: Water and sanitation: [86]; Hygiene: [15]. Results are available in these studies for water and hygiene with and without bias adjusted for non-blinding. The results above are presented without adjustment for non-blinding. As stated in [86], blinding and randomisation of study participants in water and sanitation interventions is often not possible and sometimes may not be desirable as blinding could negatively influence compliance and community dynamics which are important components for the adoption of interventions (page 11).

**Table 5:** Cost estimates of improved water and wastewater services, US$ per m^3^.

Cost Component	Full Cost	Minimal Cost
Opportunity cost of raw water supply	0.05	0.00 (“steal it“)
Storage and transmission to treatment plant	0.10	0.07 (minimum storage)
Treatment to drinking water standards	0.10	0.04 (simple chlorination)
Distribution of water to households	0.60	0.24 (PVC pipe)
Collection of wastewater from home and conveyance to wastewater treatment plant	0.80	0.30 (condominial sewers)
Wastewater treatment	0.30	0.15 (simple lagoon)
Damages associated with discharge of treated wastewater	0.05	0.00 (“someone else’s problem“)
Total	2.00	0.80

Reproduced with permission from [129]. Discount rate used is 6%. Using a 3% discount rate, the total cost is US$1.80/m^3^ at full cost and US$0.70/m^3^ at minimal cost.

**Table 6:** Benefits of improved drinking water supply and sanitation.

Benefit	Water	Sanitation
Health: burden of disease	Averted cases of diarrhoeal disease; Reduced malnutrition, enteropathy, and malnutrition-related conditions (stunting) Less dehydration from lack of access to water; Less disaster-related health impacts	Averted cases of diarrheal disease; Averted cases of helminths, polio, and eye diseases; Reduced malnutrition, enteropathy, and malnutrition-related conditions (stunting); Less dehydration from insufficient water intake due to poor latrine access; Less disaster-related health impacts
Health: economic savings	Costs related to diseases such as health care, productivity losses and premature mortality	Costs related to diseases, such as health care, productivity losses, and premature mortality
Convenience time savings	Saved travel and waiting time for water collection	Saved travel and waiting time from having nearby private toilet
Educational benefits	Improved educational levels due to higher school enrolment and attendance rates from school water; Higher attendance and educational attainment due to improved health	Improved educational levels due to higher school enrolment and attendance rates from school sanitation; Higher attendance and educational attainment due to improved health
Social benefits	Leisure and non-use values of water resources and reduced effort of averted water hauling and gender impacts	Safety, privacy, dignity, comfort, status, prestige, aesthetics, gender impacts
Water access benefits	Pretreated water at lower costs leads to averted treatment costs for households	Less pollution of water supply and hence reduced water treatment costs
Reuse		Soil conditioner and fertilizer; Energy production; Safe use of wastewater
Economic impacts	Incomes from more tourism and business investment; Employment opportunity in water provision; Rise in value of property	Incomes from more tourism and business investment; Employment opportunity in sanitation supply chain; Rise in value of property

Source: adapted from [63,84].

**Table 7:** Benefit-Cost Ratios from Global Studies.

Study and Intervention	Benefit-Cost Ratio
*Whittington et al. (2009)—modeled approach*	
Networked water and sewerage services	0.65
Deep borehole with public hand pump	4.64
Household water treatment (bio-sand filters)	2.48
Total sanitation campaign (South Asia)	3.00
*Hutton (2013)—modeled approach*	
Improved water supply (JMP definition)	2.00
Improved sanitation (JMP definition)	5.50

Source: [129,132]. All studies include the value associated with health and convenience time savings.

## Supplementary Materials

The following are available online at www.mdpi.com/1660-4601/13/6/536/s1, Table S1. Literature Presenting Damage Costs of Poor Water, Sanitation and Hygiene. Table S2. Cost-benefit studies on water, sanitation and hygiene. Table S3. Cost-effectiveness studies on water, sanitation and hygiene.

## Abbreviations

The following abbreviations are used in this manuscript:

BAT	Bottleneck analysis tool
CATS	Community approach to total sanitation
CCT	Conditional-cash transfer
CDD	Community driven development
CEA	Cost-effectiveness analysis
CI	Confidence interval
CLTS	Community-led total sanitation
DALY	Disability-adjusted life-year
DHS	Demographic and Health Survey
GDP	Gross domestic product
HLY	Healthy life-year
HWTS	Household water treatment and storage
HWWS	Handwashing with soap
JMP	WHO/UNICEF Joint monitoring programme
MDG	Millennium Development Goal
MHM	Menstrual hygiene management
OBA	Output-based-aid
OECD	Organisation for Economic Co-operation and Development
RBA	Results-based approach
SDG	Sustainable development goal
SPA	Service provision assessment
STH	Soil-transmitted helminth
UN	United Nations
UNHCR	Office of the United Nations High Commissioner for Refugees
UNICEF	United Nations International Children’s Emergency Fund
WASH	Water, sanitation, and hygiene
WHO	World Health Organization
WTP	Willingness-to-pay
