# Supplementary material for: The Knowledge Base for Achieving the Sustainable Development Goal Targets on Water Supply, Sanitation and Hygiene
Source: Int J Environ Res Public Health. 2016 May 27;13(6):536. doi: 10.3390/ijerph13060536 (PMC4923993; doi:10.3390/ijerph13060536)
Supplement: Supplementary file 1 [file ijerph-13-00536-s001.pdf]

Supplementary Materials: The Knowledge Base for Achieving the Sustainable Development Goal Targets on Water Supply, Sanitation and Hygiene

Guy Hutton and Claire Chase

Table S1. Literature Presenting Damage Costs of Poor Water, Sanitation and Hygiene.

Country (Year of Study)	Cost per Capita	Total Cost (Million)	Cost as % of GDP	Included Damage Costs	Ref. No.
Economics of Sanitation Initiative (World Bank/Water and Sanitation Program)					
India (2011)	US$49.0	US$54,000	6.4%	Poor sanitation and hygiene: health (diarrheal disease, hepatitis A and E, helminths, trachoma and infectious skin diseases), water resources, access time, tourism	[1]
Bangladesh (2011)	US$29.6	US$4200	6.3%		[2]
Pakistan (2011)	US$35.6	US$5700	3.9%		[3]
Cambodia (2008)	US$32.4	US$448	7.2%		[4]
Indonesia (2009)	US$28.6	US$6344	2.3%		[5]
Lao PDR (2009)	US$34.4	US$193	5.6%		[6]
Philippines (2008)	US$16.8	US$1412	1.5%		[7]
Vietnam (2008)	US$9.3	US$780	1.3%		[8]
Mongolia (2010)	US$10.0	US$26	0.5%	Poor sanitation and hygiene: health and access time	[9]
Sub-Saharan Africa (2008)	US$11.2	US$5500	2.0%		[10]
Cost of Environmental Degradation Studies (World Bank/Mediterranean Environmental Technical Assistance Program)					
Syria (2001)	US$9.7 [a]	US$158	0.9%	Contaminated water resources: diarrheal disease, water management and access	[11]
Morocco (2000)	US$14.6 [a]	US$420	1.2%		[12]
Iran (2002)	US$4.4 [a]	US$3200	2.2%		[13]
Jordan (2002)	US$18.9 [a]	US$100	1.2%		[14]
Tunisia (1999)	US$10.6 [a]	US$102	0.6%		[15]
Lebanon (1999)	US$48.6 [a]	US$175	1.1%		[15]
Egypt (1999)	US$9.1 [a]	US$630	1.0%		[16]
Algeria (1999)	US$23.5 [a]	US$730	1.5%		[17]
Country Environmental Analyses (World Bank/Environment Department)					
Peru (2003)	US$25.2 [a]	US$666	1.1% [a]	Inadequate WASH, range of health impacts and water boiling costs	[18]
Ghana (2004)	US$8.6	US$180	2.1%	Inadequate WASH, diarrheal diseases only	[19]
Senegal (2005)	US$3.2 [a]	US$37	0.4% [a]		[20]
Nigeria (2004)	US$4.0 [a]	US$618	1.3%		[21]
Nepal (2005)	US$3.2 [a]	US$89	1.2% [a]		[22]
Pakistan (2003)	US$12.9 [a]	US$1972 [a]	1.8%	Inadequate WASH, diarrheal and typhoid mortality and morbidity (time loss), cost of bottled water, water boiling costs, cost of hospitalization and medication due to diarrhoea and typhoid	[23]
Bangladesh (2002)	US$4.2 [a]	US$528 [b]	1.1% [b]	Inadequate WASH, range of health impacts	[24]
Guatemala (2006)	US$37.2 [a]	US$484 [a]	1.6%	Inadequate WASH, diarrheal diseases only	[25]
Egypt (2003)	US$13.1 [a]	US$911[a]	1.8%	Inadequate WASH, health costs including morbidity and treatment, other impacts such as loss in fisheries, agriculture/infrastructure losses	[26]
Tunisia (1999)	US$0.0	US$129 [a]	0.6%	Environmental damage due to water, including health costs of water-related diseases	[27]
El Salvador (2005)	-	-	1%	Inadequate WASH, health costs of waterborne diseases	[28]
Honduras (2007)	US$13.7 [a]	US $97 [a]	1%	Inadequate WASH, mortality in children, morbidity in children and adults, bottled water consumption, water chlorination, and water boiling	[29]
Philippines (2007)	US$14.7 [a]	US$1250	0.9% [a]	Inadequate WASH, range of health impacts, including malnutrition	[30]

Table S1. *Cont.*

**Other Studies**					
Pakistan (2005)<br>Ghana (2005)			5.0%<br>5.2%	Inadequate WASH health impacts, including effects on malnutrition	[31]<br>[31]
China (2003)	US$6.2 [a]	US$1200	0.6% [a]	Water pollution health costs	[32]
India (1995)	US$6.1 [a]	US$5710	1.6% [a]	Water pollution urban and rural health impacts, especially diarrhoeal diseases	[33]
India (2009)	US$9.3 [a]	US$11,103 [a]	0.8%	Inadequate WASH, environmental damage costs	[34]
Peru (1990)	US$7.4 [a]	US$200	0.4% [a]	Cholera epidemic: health, tourism, fish exports	[35]
Africa (2007)	-	US$73	-	Cholera health cost (110,000 cases)	[36]
India (2011)	US$9.1 [c]	-	-	Cost of diarrhoeal illness per urban slum household in Mumbai, including direct health care costs; avoidance costs such as extra water, kerosene and toilet fees; lost wages from income; and homemaker's productivity loss	[37]
Tanzania (2009)	US$103.2	-	-	Mean cost of illness per cholera episode in Zanzibar, including public fixed and variable treatment costs, and private direct and indirect costs	[38]
Bangladesh (2011)	US$30.40 [d]	-	-	Cost of illness per cholera episode in Dhaka, including direct medical and non-medical costs ($7.40) and indirect costs due to patient's and caregiver's income loss ($23)	[39]
Bangladesh (2011)<br>India (2011)<br>Pakistan (2011)	US$1.82 [d]<br>US$3.33 [d]<br>US$6.47 [d]	-	-	Mean cost per episode including direct medical costs, direct non-medical costs and productivity losses	[40]

Note on the initiatives: Economics of Sanitation Initiative (ESI) studies have been implemented by the Water and Sanitation Program (World Bank) in over 35 countries of Latin America and the Caribbean, East Asia and Pacific, South Asia, and Sub-Saharan Africa. These studies estimated the costs of poor sanitation, including both health and non-health impacts (access time, costs of accessing safe water, impacts on tourism). Costs of environmental degradation studies were implemented by the Mediterranean Environmental Technical Assistance Program (METAP) of the World Bank in eight Mediterranean countries from 1999 to 2002. Country environmental analyses have been conducted by the Environment Department (World Bank) in over 20 countries since 2003, and estimated the health costs of poor water and sanitation. [a] Estimated based on total impact, using exchange rates, population and GDP values from year of estimate; [b] Average savings of 15 scenarios, representing costs as % of GNI; [c] Estimate based on cost per household, using the average exchange rate for the study period, July 2011 (1 USD = 45 INR); [d] Represents cost of illness per household. Ref. No.—reference number.

Table S2. Cost-benefit studies on water, sanitation and hygiene.

Country (Setting)	Interventions Evaluated	Benefits Included	Economic Return per Currency Unit Spent	Ref. No.
**Water Supply**				
Global and regional * (2000)	Improved water supply (access)	Time savings, health (diarrhea)	11.5	[41]
	Improved water supply and household treatment		15.0	
Global and regional * (2000)	Improved water supply (access)	Time savings, health (diarrhea)	4.9	[42]
	Improved water supply and household treatment		6.3	
Global (2006)	Borehole and Public Hand Pump	Time savings, water quantity, health (diarrhea)	3.4	[43]
	Biosand filters for point-of-use water treatment	Health (diarrhea)	2.9	

	Large multi-purpose dams in Africa	Hydropower, irrigation, carbon offsets, flood prevention	2.5	
China, Henan Province	Central water supply system	Health benefits	4.4	[44]
Multi-country (2012)	Chlorination	Health benefits (morbidity and mortality reduction), time savings, Esthetic benefits	3.5	[45]
	Biosand filters		5.7	
Pakistan, Rural Abbottabad (2011)	Improved water supply	Time savings	0.43	[46]
Global (2010)	Improved drinking water sources (universal access)	Time savings, health (direct and indirect)	2.0	[47]
South Africa (1998)	Improved drinking water	Time savings, health costs, education benefits	3.1	[48]
**Sanitation**				
Global (2010)	Improved sanitation (universal access)		5.5	[47]
Global (2008)	Community-led total sanitation (CLTS)	Time savings, health (diarrhea)		[43]
Global and regional study * (2004)	Basic sanitation	Time savings, health (diarrhea)	6.6	[49]
Multi-country (model year, 2012)	Total Sanitation	Time savings, health (diarrhea)	2.2	[45]
Indonesia, Surabaya (2001)	DEWATS + ecological sanitation	User fees; health costs; productivity	1.1	[50]
	DEWATS + biogas		0.92	
	STP		0.66	
China (Qing, Beijing) (2007)	Decentralized wastewater treatment and reuse	Project revenue, water saving	3.0	[51]
Philippines, San Fernando city (2006)	UDDT (light materials)	Willingness to pay	0.86	[52]
	UDDT(concrete structure)		0.54	
	Pit latrine		1.02	
	Flush toilet to septic tank		0.36	
Uganda, Kabale (2007)	UDDT	Health, environmental, reuse	NPV = −US$345 to +US$111	[53]
	VIP		NPV = −US$124 to −US$492	
	Sewerage		NPV = −US$890	
South Africa, eThekwini (2007)	UDDT	Health, environmental, reuse	NPV = −US$1518	[53]
	VIP		NPV = −US$1148	
	Sewerage		NPV = −US$1578	
Burkina Faso, Ouagadougou (2007)	UDDT	Health, environmental, reuse	NPV = −US$396 to −US$560	[53]
	VIP		NPV = −US$842 to −US$380	
	Sewerage		NPV = −US$1055	
Pakistan, Rural Abbottabad (2011)	Sanitation	Time savings, health (diarrhea)	1.04	[54]
Cambodia (2008)	Wet pit latrines, rural areas	Health (diarrhea, helminthes, trachoma, malnutrition), time savings, water costs averted, excreta reuse	2.8	[55,56]
Indonesia (2008)			7.0	
Lao PDR (2008)			8.2	
Philippines (2008)			7.8	
Vietnam (2008)			8.0	
China (2009)			6.2	
**Hand Washing**				
Multi-country (2012)	Hand washing	Health (diarrhea)	2.6	[45]
Global and regional (2000)	Water and sanitation	Health; VSL; productivity, time savings	6.0	[42]
Africa-wide, Uganda, Rwanda, Ethiopia (2006)	Integrated biogas, latrine and hygiene programme	Fuel, health, productivity, VSL, forest, greenhouse gases, time, lighting	>4.5	[54]

China (rural areas of East Fujian)	Drinking water, health education and community outreach (environmental interventions)	Health benefits	4.9–6.5	[57]

Key: VSL—value-of-statistical-life; VIP—ventilated improved pit latrine; STP—septage treatment facility; UDDT—urine-diverting dry toilet. * For illustrative purposes, Africa epidemiological stratum E (AFR-E) is shown her. Ref. No.—reference number. Economic return per currency unit spent is the benefit-cost ratio (BCR). For example, if one United States Dollar is spent on water or sanitation, the BCR is the number of United States Dollars return on that investment.

Table S3. Cost-effectiveness studies on water, sanitation and hygiene.

Country (Setting)	Interventions Evaluated	Health Benefits Included	Cost per Death Averted	Cost per DALY Averted	Cost per Case Averted	Ref. No.
**Drinking Water**						
Rural Uganda (2004)	Household disinfection and storage for HIV people	Diarrhea		US$1252	US$5.2	[58]
Kenya (2009)	Point of use water filters for HIV infected adults	Diarrhea	US$3400	US$121	US$1.3	[59]
South Africa (n.d.)	Point of use water filters	Diarrhea, general population		US$84		[48]
		Diarrhea, children		US$47		
	Centralized water treatment system	Diarrhea, general population		US$466		
		Diarrhea, children		US$141		
Global and regional study * (2005)	Source-based protection	Diarrhea		US$123		[60]
	Household chlorination	Diarrhea		US$53		
	Household filtration	Diarrhea		US$142		
	Household solar disinfection	Diarrhea		US$61		
	Household flocculation	Diarrhea		US$472		
Global and regional study * (2000)	Household water treatment	Diarrhea		US$24		[61]
Global and regional (1996)	Safe water supply	WASH diseases	US$1,000 (SSA) to US$23,000 (China)			[62]
Global (generalized) (2006)	Hand pump or stand post	Diarrhea		US$94		[63]
	House connection	Diarrhea		US$223		
	Water sector regulation, surveillance, advocacy	Diarrhea		US$47		
**Sanitation**						
Afghanistan (Kabul) (1999)	Latrine improvement (construction or rehabilitation)	Diarrhea	US$3,436			[64]
Global (2004)	Basic sanitary latrines	Diarrhea		<US$270		[63]
	Sanitation promotion only	Diarrhea		US$11.15		
Global and regional (1996)	Safe sanitation facility	WASH diseases	US$3,000 (SSA) to US$23,000 (China)			[62]

Cambodia (2008)	Wet pit latrines, rural areas (cost-effectiveness of other technologies and urban areas presented in [55])	Diarrhea, helminthes, malnutrition and disease related to malnutrition	US$16,377	US$433	US$12.3	[55,56]
Indonesia (2008)			US$45,031	US$786	US$4.7	
Lao PDR (2009)			US$18,503	US$953	US$7.5	
Philippines (2008)			US$56,799	US$2996	US$10.7	
Vietnam (2008)			US$6965	US$756	US$8.0	
Yunnan Province, China (2009)			US$18,921	US$1,039	US$9.3	
**Hygiene**						
Burkina Faso (Bobo-Dioulasso) (1999)	Health education for mothers	Health (diarrhea children under 5)	US$51			[65]
Bangladesh (rural) (1995)	Health education	Health (intestinal parasites)				
Global and regional (1996)	Hygiene improvement	WASH diseases			US$1 spent leads to 0.3% reduction in helminthes	[66,67]
Global (1996)	Social marketing and education (SME) on hygiene alone	Child diarrhea	US$1520	US$44	US$6.5	[68]
	SME on top of existing hardware		US$689	US$20	US$2.9	[68]
	SME and hardware together		US$14,253	US$413	US$60.1	[68]
Global (2004)	Hygiene interventions	Diarrhea		US$3.4		[63]
**Combined Water, Sanitation and Hygiene Interventions**						
Guinea (1994)	Latrines and safe water	Health (diarrhea children under 5)		US$343 (per life year saved)		[69]
Global and regional (2000)	Water and sanitation	Health, VSL, productivity, time savings		-	US$534	[69]
Global (1996)	Software interventions added to existing hardware	Health (diarrhea children under 5)	US$689	US$20		[68]
	Hardware and software combined		US$14,253	US$413		
	Hardware only		US$39,720	US$1152	US$169	
	Software only			US$44		

* For illustrative purposes, Africa epidemiological stratum E (AFR-E) is shown here. SSA—Sub-Saharan Africa; VSL—value-of-statistical life. Ref. No.—reference number. n.d.—no date (year of study not available).

References

1. Tyagi, A. *Economic Impacts of Inadequate Sanitation in India*; World Bank, Water and Sanitation Program: Washington, DC, USA, 2010.
2. Barkat, A. *Economic Impacts of Inadequate Sanitation in Bangladesh*; World Bank, Water and Sanitation Program: Washington, DC, USA, 2011.
3. Nishat, M. *The Economic Impacts of Inadequate Sanitation in Pakistan*; World Bank, Water and Sanitation Program: Washington, DC, USA, 2012.
4. Kov, P.; Sok, H.; Roth, S.; Chhoeun, K.; Hutton, G. *Economic Impacts of Sanitation in Cambodia*; World Bank, Water and Sanitation Program: Washington, DC, USA, 2009.
5. Napitupulu, L.; Hutton, G. *Economic Impacts of Sanitation in Indonesia*; World Bank, Water and Sanitation Program: Washington, DC, USA, 2008.
6. Hutton, G.; Larsen, B.; Leebouapao, L.; Voladet, S. *Economic Impacts of Sanitation in Lao PDR*; World Bank, Water and Sanitation Program: Washington, DC, USA, 2009.
7. Rodriguez, U.; Jamora, N.; Hutton, G. *Economic Impacts of Sanitation in the Philippines*; World Bank, Water and Sanitation Program: Washington, DC, USA, 2008.
8. Thang, P.; Tuan, H.; Hutton, G. *Economic Impacts of Sanitation in Vietnam*; World Bank, Water and Sanitation Program: Washington, DC, USA, 2008.

9. Hutton, G.; Amartuvshin, A. *Economic Impacts of Sanitation in Mongolia*; UNICEF: Ulaanbaatar, Mongolia, 2011.
10. Hickling, S.; Hutton, G. Economics of inadequate sanitation in Africa. In *Sanitation and Hygiene in Africa: Where Do We Stand? Analysis from the AfricaSan Conference, Kigali, Rwanda*; Cross, P., Coombes, Y., Eds.; IWA Publishing: London, UK, 2014.
11. Sarraf, M.; Bolt, K.; Larsen, L. *Cost Assessment of Environmental Degradation in the Syrian Arab Republic*; World Bank, Mediterranean Environmental Technical Assistance Program: Washington, DC, USA, 2004.
12. Sarraf, M.; Belhaj, M.; Jorio, A. *Cost Assessment of Environmental Degradation in the Kingdom of Morocco*; World Bank, Mediterranean Environmental Technical Assistance Program: Washington, DC, USA, 2003.
13. Sarraf, M.; Owaygen, M.; Croitoru, L. *Cost Assessment of Environmental Degradation in the Islamic Republic of Iran*; World Bank, Mediterranean Environmental Technical Assistance Program: Washington, DC, USA, 2005.
14. Mediterranean Environmental Technical Assistance Program (METAP). *Hashemite Kingdom of Jordan: Cost of Environmental Degradation*; World Bank: Washington, DC, USA, 2005.
15. Sarraf, M.; Larsen, B.; Owaygen, M. *Cost of Environmental Degradation—The Case of Lebanon and Tunisia*; World Bank, Mediterranean Environmental Technical Assistance Program: Washington, DC, USA, 2004.
16. Sarraf, M.; Larsen, B. *Cost Assessment of Environmental Degradation in the Arab Republic of Egypt*; World Bank, Mediterranean Environmental Technical Assistance Program: Washington, DC, USA, 2004.
17. Sarraf, M. *Cost Assessment of Environmental Degradation in the Democratic Republic of Algeria (in French)*; World Bank, Mediterranean Environmental Technical Assistance Program; Ministère de l'Aménagement du Territoire et de l'Environnement: Washington, DC, USA, 2002.
18. Shnchez-Triana, E.; Awe, Y.; Poveda, R.; Urmtia Vhsquez, C.; Cherres, M.; Alva, A.; Reuben, W.; Bortman, M.; Hatziolos, M.; Pillai, P.; *et al. Environmental Sustainability: A Key to Poverty Reduction in the Republic of Peru*; World Bank, Environmentally and Socially Sustainable Development Department: Washington, DC, USA, 2007.
19. Fiadjoe, Y.; Kobayashi, H.; Loayza, F.; Burckhart, B.; Marchisio, M.; Green, T.; Larsen, B.; Skjelvik, J.; von Thadden, S. *Country Environmental Analysis, Ghana*; World Bank, Environment and Natural Resources Department: Washington, DC, USA, 2007.
20. Kristensen, P.; Bennouna, T.; Danyo, S.; Pillai, P.; Prevost, Y.; Ruta, G.; Sene, M. *Country Environmental Analysis, Senegal*; World Bank, Sustainable Development Department: Washington, DC, USA, 2008.
21. Margulis, S.; Pillai, P.; Ruta, G.; Chee, N.; Olojoba, A.; Akapa, L.; Papka, P.; Akpokodje, J.; Okorie, A. *Rapid Country Environmental Analysis, Nigeria*; World Bank, Environment, Rural and Social Development: Washington, DC, USA, 2006.
22. Rahill, B.; Nishida, J.; Tenzing, T.; Kumar, P.; Pilapitiya, S.; Acharya, A.; Ghimire, D.; Shrestha, T.; Ghosh, V. *Country Environmental Analysis, Nepal*; World Bank, Sustainable Development Department: Washington, DC, USA, 2007.
23. Martin, P. *Pakistan Strategic Country Environmental Assessment*; World Bank: Washington, DC, USA, 2006.
24. Khaliquzzaman, M. *Bangladesh Country Environmental Analysis, Technical Annex: Health Impacts of Air and Water Pollution in Bangladesh*; World Bank, South Asia Environment and Social Development Unit: Washington, DC, USA, 2006.
25. Belausteguigoitia, J. *Republic of Guatemala Country Environmental Analysis Addressing the Environmental Aspects of Trade and Infrastructure Expansion*; 36459-GT; World Bank, Environmentally and Socially Sustainable Development Department, Latin America and the Caribbean Region: Washington, DC, USA, 2006.
26. Arif, S. *Arab Republic of Egypt Country Environmental Analysis (1992–2002)*; 31993-EG; World Bank, Water, Environment, Social and Rural Development Department, The Middle East and North Africa Region: Washington, DC, USA, 2005.
27. Arif, S. *Tunisia Country Environmental Analysis (1992–2003)*; 25966-TN; World Bank, Water, Environment, Rural and Social Development, Middle East and North Africa Region: Washington, DC, USA, 2004.
28. Belausteguigoitia, J. *Republic of el Salvador Country Environmental Analysis, Improving Environmental Management to Address Trade Liberalization and Infrastructure Expansion; 35226-SV*; World Bank, Environmentally and Socially Sustainable Development Department. Latin America and the Caribbean Region: Washington, DC, USA, 2006.
29. Strukova, E. *Honduras. Health Costs of Environmental Damage*; World Bank: Washington, DC, USA, 2007.
30. Bojö, J.; Villaluz, M. *Country Environmental Analysis, the Philippines*; World Bank, Sustainable Development Department: Washington, DC, USA, 2009.

31. Acharya, A.; Paunio, M.; Ahmed, K. *Environmental Health and Child Survival: Epidemiology, Economics and Experience*; World Bank: Washington, DC, USA, 2008.
32. World Bank. *Cost of Pollution in China. Economic Estimates of Physical Damages*; World Bank, Environmental and Social Development Unit and the PR China State Environmental Protection Administration: Washington, DC, USA, 2007.
33. Brandon, C.; Hommann, K. *The Cost of Inaction: Valuing the Economy-Wide Cost of Environmental Degradation in India*; World Bank, Asia Environment Division: Washington, DC, USA, 1996.
34. Mani, M. *India: Diagnostic Assessment of Select Environmental Challenges*; World Bank, Disaster Management and Climate Change Unit, Sustainable Development Department, South Asia Region: Washington, DC, USA, 2013.
35. Suarez, R.; Bradford, B. *The Economic Impact of the Cholera Epidemic in Peru: An Application of the Cost-If-Illness Methodology*; WASH Field Report No. 415; Water and Sanitation for Health Project: Arlington, VA, USA, 1991.
36. Kirigia, J.; Sambo, L.; Yokouide, A.; Soumbey-Alley, E.; Muthuri, L.; Kirigia, D. Economic burden of cholera in the who African region. *BMC Int. Health Hum. Rights* **2009**, *9*, doi:10.1186/1472-698X-9-8.
37. Patel, R.B.; Stoklosa, H.; Shitole, S.; Shitole, T.; Sawant, K.; Nanarkar, M.; Subbaraman, R.; Ridpath, A.; Patil-Deshmuk, A. The high cost of diarrhoeal illness for urban slum households—A cost-recovery approach: A cohort study. *Br. Med. J. Open* **2013**, *3*, e002251.
38. Schaetti, C.; Weiss, M.G.; Ali, S.M.; Chaignat, C.L.; Khatib, A.M.; Reyburn, R.; Tebbens, R.J.D.; Hutubessy, R. Costs of illness due to cholera, costs of immunization and cost-effectiveness of an oral cholera mass vaccination campaign in Zanzibar. *PLoS Negl. Trop. Dis.* **2012**, *6*, e1844.
39. Sarker, A.R.; Islam, Z.; Khan, I.A.; Saha, A.; Chowdhury, F.; Khan, A.I.; Qadri, F.; Khan, J.A. Cost of illness for cholera in a high risk urban area in Bangladesh: An analysis from household perspective. *BMC Infect. Dis.* **2013**, *13*, doi:10.1186/1471-2334-13-518.
40. Rheingans, R.; Kukla, M.; Faruque, A.S.G.; Sur, D.; Zaidi, A.K.; Nasrin, D.; Farag, T.H.; Levine, M.M.; Kotloff, K.L. Determinants of household costs associated with childhood diarrhea in 3 south Asian settings. *Clin. Infect. Dis.* **2012**, *55*, S327–S335.
41. Hutton, G.; Haller, L. *Evaluation of the Non-Health Costs and Benefits of Water and Sanitation Improvements at Global Level; WHO/SDE/WSH/04.04*; World Health Organization: Geneva, Switzerland, 2004.
42. Hutton, G.; Haller, L.; Bartram, J. Global cost-benefit analysis of water supply and sanitation interventions. *J. Water Health* **2007**, *5*, 481–502.
43. Whittington, D.; Hanemann, W.; Sadoff, C.; Jeuland, M. *Sanitation and Water. Copenhagen Consensus 2008 Challenge Paper*; Copenhagen Consensus Center: Copenhagen, Denmark, 2008.
44. Lou, H. Effectiveness evaluation and cost-effectiveness estimate for diarrhea control by environment improvement in rural area. *Zhonghua Liu Xing Bing Xue Za Zhi* **1990**, *11*, 170–174.
45. Whittington, D.; Jeuland, M.; Barker, K.; Yuen, Y. Setting priorities, targeting subsidies among water, sanitation, and preventive health interventions in developing countries. *World Dev.* **2012**, *40*, 1546–1568.
46. Sher, M.H.S.; Iqbal, S. Socio-economic analysis of the interventions aimed at improving water and sanitation condition of rural community. *Res. Hum. Soc. Sci.* **2012**, *2*, 10.
47. Hutton, G. *Global Costs and Benefits of Drinking-Water Supply and Sanitation Interventions to Reach the MDG Target and Universal Coverage*; Report No. Who/hse/wsh/12.01; World Health Organization: Geneva, Switzerland, 2012.
48. Cameron, J.; Jagals, P.; Hunter, P.; Pedley, S.; Pond, K. Economic assessments of small-scale drinking-water interventions in pursuit of MDG target 7c. *Sci. Total Environ.* **2011**, *410–411*, 8–15.
49. Hutton, G.; Haller, L.; Bartram, J. *Economic and Health Effects of Increasing Coverage of Low Cost Household Drinking Water Supply and Sanitation Interventions to Countries Off-Track to Meet MDG Target 10*; Who/sde/wsh/07.05; World Health Organization, United Nations Development Programme: Geneva, Switzerland, 2007.
50. Prihandrijanti, M.; Malisie, A.; Otterpohl, R. Cost-benefit analysis for centralized and decentralized wastewater treatment system (case study in Surabaya, Indonesia). In *Efficient Management of Wastewater: Its Treatment and Reuse in Water-Scarce Countries*; Al Baz, I., Otterpohl, R., Wendland, C., Eds.; Springer: Berlin, Germany; Heidelberg, Germany, 2008.

51. Liang, X.; van Dijk, M. Economic and Financial Analysis of decentralized Water Recycling Systems in Beijing. In Proceedings of the 3rd SWITCH Scientific Meeting, Belo Horizonte, Brazil, 30 November–4 December 2008.
52. Ignacio, J. A Comparative Benefit-Cost and Cost-Effectiveness Analysis of Ecosan and Non-Ecosan Systzems in San Fernando City, La Union, Philippines; Foundation for a sustainable society: Quezon City, Philippines, 2006.
53. Schuen, R.; Parkinson, R.; Ross, P.; Jeal, G. *Study for Economic and Financial Analysis of Ecological Sanitation in Sub-Saharan Africa*; World Bank, Water and Sanitation Program: Washington, DC, USA, 2008.
54. Renwick, M.; Sagar Subedi, P.; Hutton, G. A Cost-Benefit Analysis of National and Regional Integrated Biogas and Sanitation Programs in Sub-Saharan Africa; Winrock International: Arlington, VA, USA, 2007.
55. Hutton, G.; Rodriguez, U.; Winara, A.; Nguyen, V.; Kov, P.; Chuan, L.; Blackett, I.; Weitz, A. Economic efficiency of sanitation interventions in southeast Asia. *J. Water Sanit. Hyg. Dev.* **2014**, *4*, 23–36.
56. Hutton, G.; Rodriguez, U.; Winara, A.; Nguyen, V.; Sok Heng, S.; Kov, P.; Chuan, L.; Blackett, I.; Weitz, A. *Economic Assessment of Sanitation Interventions in Southeast Asia*; Water and Sanitation Program, The World Bank: Washington, DC, USA, 2015.
57. Xiao, S.; Lin, C.; Chen, K. Evaluation of effectiveness of comprehensive control for diarrhea diseases in rural areas of east Fujian and analysis of its cost-benefit. *Zhonghua Liu Xing Bing Xue Za Zhi* **1997**, *31*, 40–41.
58. Shrestha, R.; Marseille, E.; Kahn, J.; Lule, J.; Pitter, C.; Blandford, J.; Bunnell, R.; Coutinho, A.; Kizito, F.; Quick, R.; *et al.* Cost-effectiveness of home-based chlorination and safe water storage in reducing diarrhea among HIV-affected households in rural Uganda. *Am. J. Hyg. Trop. Med.* **2006**, *74*, 884–890.
59. Kern, E.; Verguet, S.; Yuhas, K.; Odhiambo, F.H.; Kahn, J.G.; Walson, J. Provision of bednets and water filters to delay HIV-1 progression: Cost-effectiveness analysis of a Kenyan multisite study. *Trop. Med. Int. Health* **2013**, *18*, 916–924.
60. Clasen, T.; Haller, L.; Walker, D.; Bartram, J.; Cairncross, S. Cost-effectiveness of water quality interventions for preventing diarrhoeal disease in developing countries. *J. Water Health* **2007**, *5*, 599–608.
61. Haller, L.; Hutton, G.; Bartram, J. Estimating the costs and health benefits of water and sanitation improvements at global level. *J. Water Health* **2007**, *5*, 467–480.
62. Larsen, B. Hygiene and health in developing countries: Defining priorities through cost-benefit assessments. *Int. J. Environ. Health Res.* **2003**, *13*, 37–46.
63. Cairncross, S.; Valdmanis, V. Water supply, sanitation and hygiene promotion. In *Disease Control Priorities in Developing Countries*, 2nd ed.; Jamison, D., Breman, J., Measham, A., Alleyne, G., Claeson, M., Evans, D., Jha, P., Mills, A., Musgrove, P., Eds.; Oxford University Press: New York, NY, USA, 2006.
64. Meddings, D.; Ronald, L.; Marion, S.; Pinera, J.; Oppliger, A. Cost effectiveness of a latrine revision programme in Kabul, Afghanistan. *Bull. World Health Organ.* **2004**, *82*, 281–289.
65. Borghi, J.; Guinness, L.; Ouedraogo, J.; Curtis, V. Is hygiene promotion cost-effective? A case study in Burkina Faso. *Trop. Med. Int. Health* **2002**, *7*, 960–969.
66. Mascie-Taylor, C.; Karim, R.; Karim, E.; Akhtar, S.; Ahmed, T.; Montanari, R. The cost-effectiveness of health education in improving knowledge and awareness about intestinal parasites in rural Bangladesh. *Econ. Hum. Biol.* **2003**, *1*, 321–330.
67. Mascie-Taylor, C.; Alam, M.; Montanari, R.; Karim, R.; Ahmed, T.; Karim, E.; Akhtar, S. A study of the cost effectiveness of selective health interventions for the control of intestinal parasites in rural Bangladesh. *J. Parasitol.* **1999**, *85*, 6–11.
68. Varley, R.; Tarvid, J.; Chao, D. A reassessment of the cost-effectiveness of water and sanitation interventions in programmes for controlling childhood diarrhoea. *Bull. World Health Organ.* **1998**, *76*, 617–631.
69. Jha, P.; Bangoura, O.; Ranson, K. The cost-effectiveness of forty health interventions in Guinea. *Health Policy Plan.* **1998**, *13*, 249–262.

© 2016 by the authors; licensee MDPI, Basel, Switzerland. This article is an open access article distributed under the terms and conditions of the Creative Commons by Attribution (CC-BY) license (http://creativecommons.org/licenses/by/4.0/).
